# Functional characterisation of twelve terpene synthases from actinobacteria

**DOI:** 10.3762/bjoc.19.100

**Published:** 2023-09-15

**Authors:** Anuj K Chhalodia, Houchao Xu, Georges B Tabekoueng, Binbin Gu, Kizerbo A Taizoumbe, Lukas Lauterbach, Jeroen S Dickschat

**Affiliations:** 1 Kekulé-Institute of Organic Chemistry and Biochemistry, University of Bonn, Gerhard-Domagk-Straße 1, 53121 Bonn, Germany

**Keywords:** actinomycetes, biosynthesis, enzymes, NMR spectroscopy, terpenes

## Abstract

Fifteen type I terpene synthase homologs from diverse actinobacteria that were selected based on a phylogenetic analysis of more than 4000 amino acid sequences were investigated for their products. For four enzymes with functions not previously reported from bacterial terpene synthases the products were isolated and their structures were elucidated by NMR spectroscopy, resulting in the discovery of the first terpene synthases for (+)-δ-cadinol and (+)-α-cadinene, besides the first two bacterial (−)-amorpha-4,11-diene synthases. For other terpene synthases with functions reported from bacteria before the products were identified by GC–MS. The characterised enzymes include a new *epi*-isozizaene synthase with monoterpene synthase side activity, a 7-*epi*-α-eudesmol synthase that also produces hedycaryol and germacrene A, and four more sesquiterpene synthases that produce mixtures of hedycaryol and germacrene A. Three phylogenetically related enzymes were in one case not expressed and in two cases inactive, suggesting pseudogenisation in the respective branch of the phylogenetic tree. Furthermore, a diterpene synthase for allokutznerene and a sesterterpene synthase for sesterviolene were identified.

## Introduction

**Figure 1:**
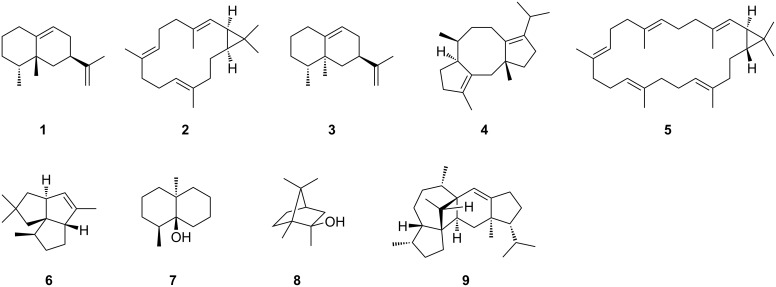
Terpenes produced by characterised terpene synthases.

Terpene synthases are remarkable enzymes that can convert acyclic and achiral oligoprenyl pyrophosphates into terpene hydrocarbons or alcohols of high structural complexity. These enzymatic reactions are initiated by ionisation of the substrate either through diphosphate abstraction (for type I terpene synthases) or protonation of the substrate (type II terpene synthases). The resulting cationic species can then react in a cascade reaction via a series of cationic intermediates involving cyclisations, hydride or proton shifts, and skeletal rearrangements. During the past decades numerous enzymes have been characterised from all branches of life. Only considering type I terpene synthases, after the identification of the 5-*epi*-aristolochene (**1**) synthase from *Nicotiana tabacum* [1] and the casbene (**2**) synthase from *Ricinus communis* [2] (Figure 1), hundreds of plant terpene synthases have been identified [3–4], including terpene synthases of microbial type [5]. Also many fungal terpene synthases are known that can either be monofunctional as in case of the aristolochene (**3**) synthases from *Aspergillus terreus* [6] and *Penicillium roqueforti* [7], or they may be bifunctional and composed of two domains. In these enzymes a prenyltransferase domain catalyses the formation of an oligoprenyl pyrophosphate precursor from dimethylallyl pyrophosphate (DMAPP) and isopentenyl pyrophosphate (IPP) that is subsequently cyclised by the terpene synthase domain. The first discovered example from this class is the fusicoccadiene (**4**) synthase from *Phomopsis amygdali* [8], and even triterpenes such as macrophomene (**5**) can be generated by these bifunctional enzymes [9]. After cloning of the gene for pentalenene (**6**) synthase from *Streptomyces exfoliatus* [10], many bacterial terpene synthases have been identified [11], including enzymes for the non-canonical compounds geosmin (**7**) [12] and 2-methylisoborneol (**8**) [13]. Recent developments also revealed the presence of sesterterpene synthases in bacteria exemplified by the enzymes for sesterviridene (**9**) in *Kitasatospora viridis* [14–16]. Only few terpene synthases have been characterised from other organisms, including enzymes from insects [17], octocorals [18–19], red algae [20–21], and amobae [22–23]. Despite these previous efforts, for many known terpenes still no terpene synthases catalysing their formation have been reported. Here, we report on the discovery and functional characterisation of four sesquiterpene synthases from actinomycetes with novel functions, in addition to several actinomycete terpene synthases for which functional homologs have been identified before.

## Results and Discussion

### Phylogenetic analysis

**Figure 2:**
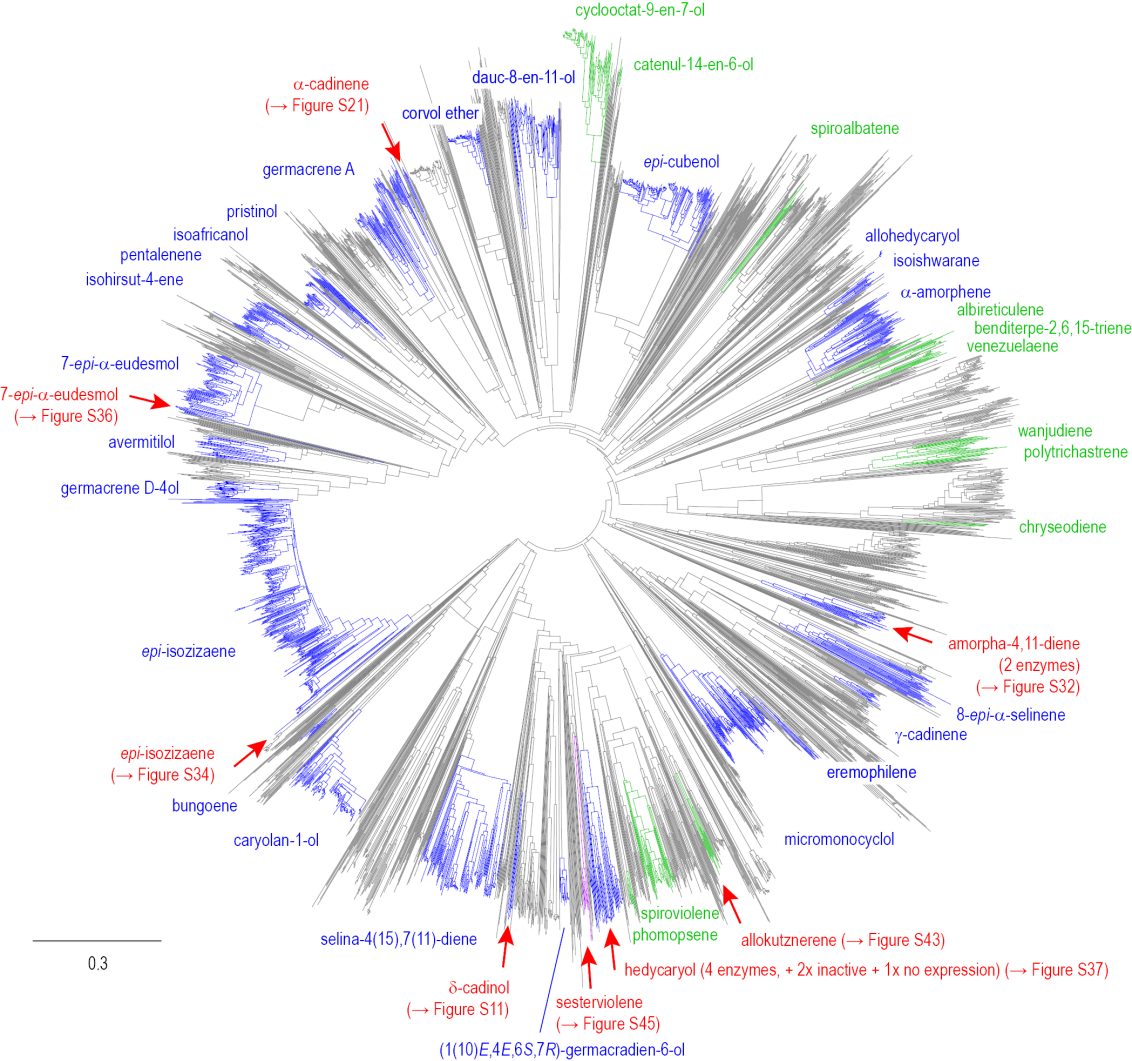
Phylogenetic tree constructed from the amino acid sequences of 4018 terpene synthase homologs. Blue branches indicate groups of homologous sesquiterpene synthases, green branches indicate groups of homologous diterpene synthases, and purple branches indicate groups of homologous sesterterpene synthases from which at least one representative was functionally characterised. The red arrows highlight enzymes characterised in this study (cf. the indicated Figures of Supporting Information File 1 for a detailed view). The scale bar indicates substitutions per site.

A phylogenetic tree was constructed from 4018 bacterial terpene synthase homologs (Figure 2). In this tree all branches of homologous enzymes for which at least one representative was functionally characterised are shown in blue, whereas the uncharacterised enzymes are shown in grey, revealing that the functions of still many terpene synthase homologs are unknown. Some of the largest branches in this tree represent the homologs of *epi*-isozizaene synthase from *Streptomyces coelicolor* [24], caryolan-1-ol synthase from *Streptomyces griseus* [25], selina-4(15),7(11)-diene synthase from *Streptomyces pristinaespiralis* [26], spiroviolene synthase from *Streptomyces violens* [27], micromonocyclol synthase from *Micromonospora marina* [28], α-amorphene synthase from *Streptomyces viridochromogenes* [29–30], *epi*-cubenol synthase from *S. griseus* [31], germacrene A synthase from *M. marina* [32], and 7-*epi*-α-eudesmol synthase from *S. viridochromogenes* [29–30]. In order to expand the knowledge about terpene synthase catalysis, fifteen uncharacterised terpene synthase homologs as listed in Table 1 were selected for further studies from different branches of the tree (indicated by red arrows in Figure 2). The genes coding for all fifteen enzymes were amplified by PCR from genomic DNA, cloned and expressed in *Escherichia coli*. The purified recombinant proteins (Figure S1, Supporting Information File 1) were used in test incubations with geranyl pyrophosphate (GPP), farnesyl pyrophosphate (FPP), geranylgeranyl pyrophosphate (GGPP) and geranylfarnesyl pyrophosphate (GFPP).

**Table 1:** Terpene synthase homologs characterised in this study.

entry	organism	accession no.	(main) product

1	*Kutzneria kofuensis* DSM 43851	MBB5895433	(+)-δ-cadinol^a^
2	*Streptomyces jumonjiensis* NRRL 5741	WP_153520876	(+)-α-cadinene^a^
3	*Streptomyces lavendulae* NRRL B-2774	WP_078950427	(−)-amorpha-4,11-diene^a^
4	*Streptomyces subrutilus* ATCC 27467	WP_150516140	(−)-amorpha-4,11-diene^a^
5	*Nocardia brevicatena* NBRC12119	WP_086008896	*epi*-isozizaene^a^
6	*Streptomyces flavidovirens* DSM 40150	WP_028812116	7-*epi*-α-eudesmol^a^
7	*Streptomyces sclerotialus* NRRL ISP-5269	WP_030615021	hedycaryol^a^
8	*Streptomyces catenulae* NRRL B-2342	WP_051739595	hedycaryol^a^
9	*Streptomyces ficellus* NRRL 8067	WP_156694351	hedycaryol^a^
10	*Streptomyces morookaense* DSM 40503	WP_171082395	germacrene A^a^
11	*Streptomyces subrutilus* ATCC 27467	WP_150522245	no expression
12	*Streptomyces natalensis* NRRL B-5314	WP_037793252	inactive
13	*Streptomyces violens* NRRL ISP-5597	WP_030249874	inactive
14	*Kutzneria kofuensis* DSM 43851	WP_184867163	allokutznerene^b^
15	*Streptomyces* sp. Tü 2975	WP_159685978	sesterviolene^c^

^a^From FPP as substrate (sesquiterpene synthase). ^b^From GGPP as substrate (diterpene synthase). ^c^From GFPP as substrate (sesterterpene synthase).

### Sesquiterpene synthases

**Figure 3:**
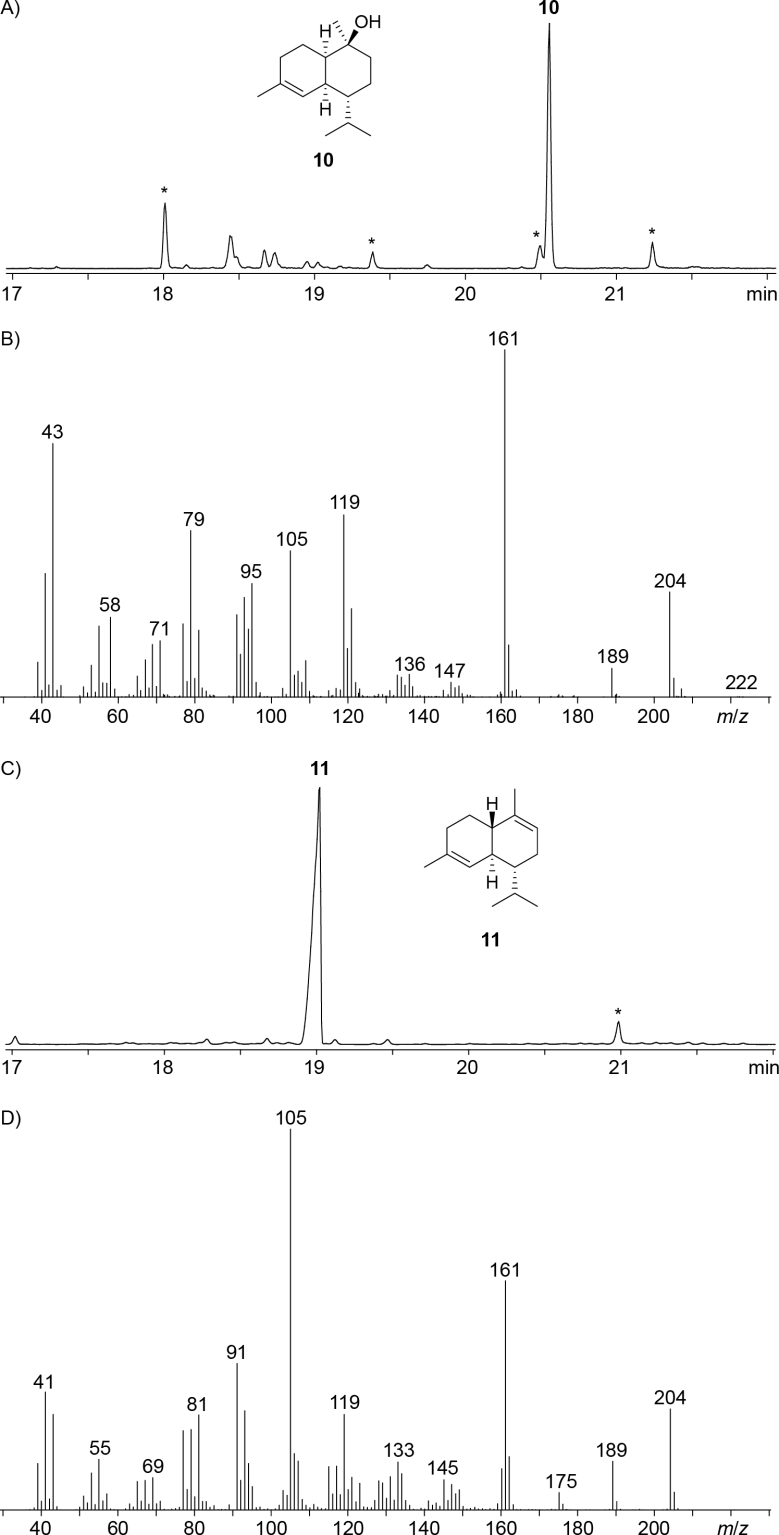
A) Total ion chromatogram of the products obtained from FPP with KkdCS, B) EI mass spectrum of **10**, C) total ion chromatogram of the products obtained from FPP with SjaCS, D) EI mass spectrum of **11**. Asterisks indicate acyclic products and contaminants such as plasticisers.

The enzyme from *K. kofuensis* (Table 1, entry 1) exhibited all highly conserved motifs required for functionality including the aspartate-rich motif (^83^DDAYCD) and the NSE triad (^223^NDIASYYKE, Figure S2, Supporting Information File 1). The closest characterised terpene synthase with an amino acid sequence identity of 25% is the (1(10)*E*,4*E*,6*S*,7*R*)-germacradien-6-ol synthase from *Streptomyces pratensis* [33]. The recombinant enzyme efficiently converted FPP into one sesquiterpene alcohol whose electron ionisation (EI) mass spectrum suggested the structure of δ-cadinol (**10**) by comparison to a mass spectrum included in the NIST Standard Reference Database (Figure 3A and 3B). Only minor amounts of acyclic products were obtained from GPP (myrcene, ocimene, linalool) and GGPP (β-springene), while GFPP was not accepted. A preparative scale incubation of FPP (80 mg, 185 μmol) allowed for the isolation of **10** (5.5 mg, 25 μmol, 14%) for structure elucidation through NMR spectroscopy (Table S2, Figures S3–S10, Supporting Information File 1), confirming the structure of δ-cadinol. The optical rotation of [α]_D_^25^ = +95.9 (*c* 0.55, CH_2_Cl_2_) pointed to the same enantiomer as is known from the plants *Pinus sibirica* ([α]_D_^20^ = +118.4) and *Torreya nucifera* ([α]_D_^18^ = +118.6) [34], and from the fungus *Xylobolus frustulatus* ([α]_D_^25^ = +99.9 (*c* 0.6, CHCl_3_)) [35]. This finding is rather unusual, as more and more cases were recently identified in which sesquiterpenes from bacteria showed an enantiomeric relationship to plant compounds [36]. The enzyme from *K. kofuensis* represents the first terpene synthase for the biosynthesis of **10** and was thus identified as *Kutzneria kofuensis* (+)-δ-Cadinol Synthase (KkdCS). A few closely related enzymes from other actinomycetes with a pairwise identity of 69% may also function as (+)-δ-cadinol synthases (Figure S11, Supporting Information File 1).

The enzyme from *S. jumonjiensis* (Table 1, entry 2) showed the fully established conserved motifs including the aspartate-rich region (^83^DDVRSE) and the NSE triad (^225^NDIHSYEKE, Figure S12, Supporting Information File 1) and its closest characterised relative is with 32% identity the germacrene A synthase from *M. marina* [32]. The incubation with GPP resulted in minor amounts of acyclic compounds (myrcene, ocimene, linalool), while FPP gave a high yield of α-cadinene (**11**) (Figure 3C and 3D), and GGPP and GFPP were not accepted as substrate. For verification of the GC–MS-based identification the product was isolated from a preparative scale incubation of FPP (80 mg, 185 μmol) to obtain pure **11** (1.3 mg, 6.4 μmol, 3.5%). Structure elucidation by NMR spectroscopy confirmed the identity of the enzyme product α-cadinene (Table S3, Figures S13–S20, Supporting Information File 1). The optical rotation of [α]_D_^25^ = +60.0 (*c* 0.015, C_6_D_6_) indicated the opposite enantiomer as in the plant *Humulus lupulus* ([α]_D_^24^ = −62.4 (*c* 0.868, CHCl_3_)) [37]. (+)-δ-Cadinene synthases have been described from *Gossypium arboreum* [38] and *Gossypium hirsutum* [39], a (−)-δ-cadinene synthase was identified in *Streptomyces clavuligerus* [40], and (−)-γ-cadinene synthases are known from the termite associated fungus *Termitomyces* sp. [41] and the bacterium *Chitinophaga pinensis* [29–30], but no α-cadinene synthase has been reported to date. The enzyme newly described here was designated as *Streptomyces jumonjiensis* (+)-α-Cadinene Synthase (SjaCS). A few more enzymes with a pairwise identity of 83% are observed in other actinomycetes that likely also function as (+)-α-cadinene synthases (Figure S21, Supporting Information File 1).

**Figure 4:**
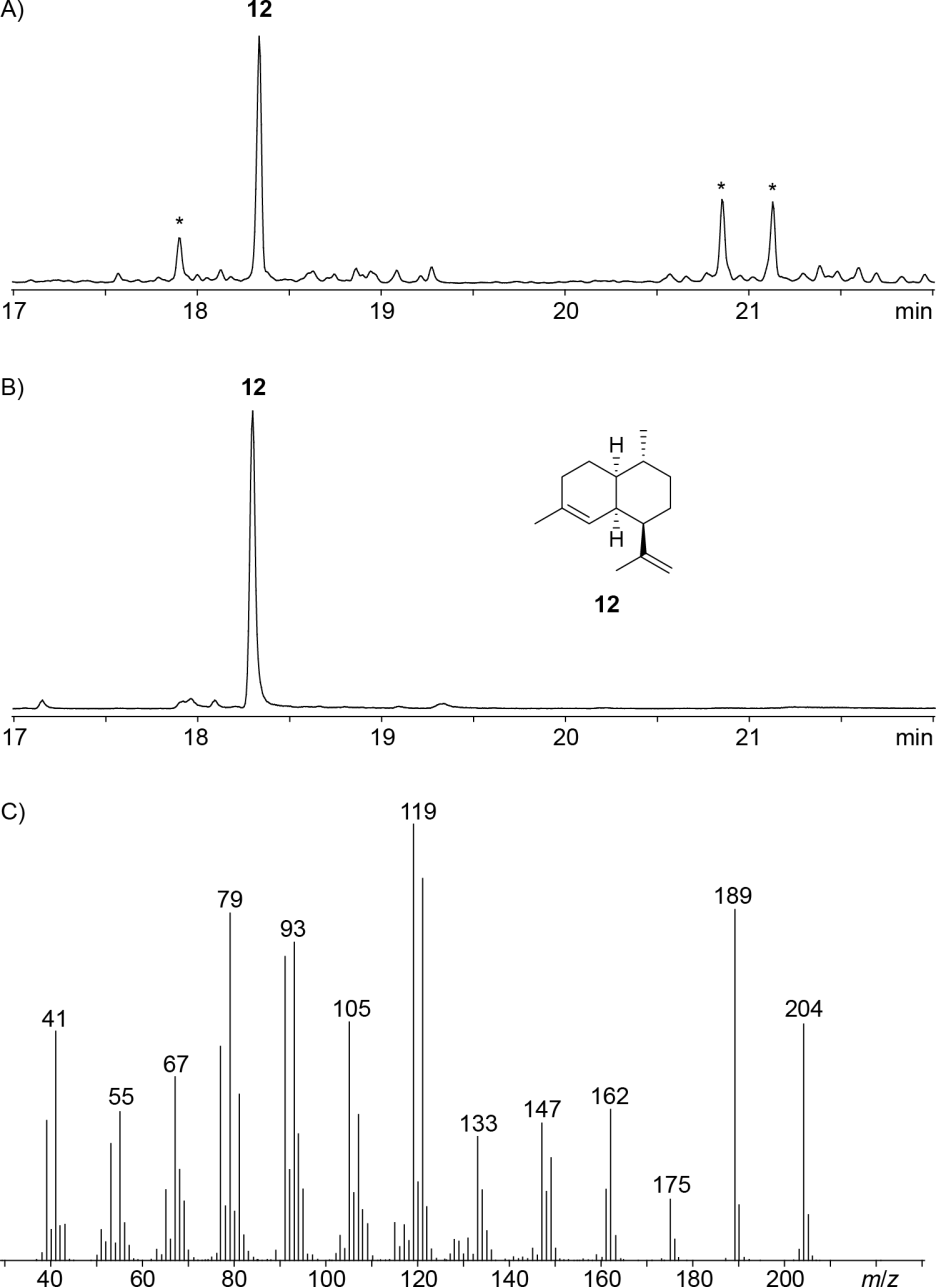
Total ion chromatograms of the products obtained from FPP A) with SlADS and B) with SsADS, C) EI mass spectrum of **12**. Asterisks indicate acyclic products and contaminants such as plasticisers.

The enzyme from *S. lavendulae* (Table 1, entry 3) exhibited all highly conserved motifs including the aspartate-rich sequence (^83^DDQHD) and the NSE triad (^226^NDVFSLPKE, Figure S22, Supporting Information File 1). The closely related homolog from *S. subrutilus* (Table 1, entry 4) showed the same sequences for these motifs (Figure S23, Supporting Information File 1). Both enzymes are distant from all previously characterised terpene synthases and show a sequence identity of only 25% and 28%, respectively, to their closest characterised homolog spiroalbatene synthase from *Allokutzneria albata* [42]. Test incubations with GPP resulted in the formation of acyclic products besides minor amounts of limonene, while GGPP and GFPP were not converted by both enzymes. With FPP both enzymes resulted in the formation of a sesquiterpene hydrocarbon that was identified by GC–MS as amorpha-4,11-diene (**12**, Figure 4). The structure of the product was confirmed through a preparative scale incubation of FPP (80 mg, 185 μmol) yielding pure **12** (1 mg, 4.9 μmol, 2.6%) for NMR spectroscopic analysis (Table S4, Figures S24–S31, Supporting Information File 1). The optical rotation of [α]_D_^25^ = –9.4 (*c* 0.64, CH_2_Cl_2_) pointed to the same enantiomer as in the plant *Viguiera oblongifolia* ([α]_D_^24^ = –8 (*c* 0.4, CHCl_3_)) [43]. A (−)-amorpha-4,11-diene synthase (ADS) is also known from *Artemisia annua* and catalyses the first committed step in the biosynthesis of artemisinin [44]. From bacteria only the α-amorphene synthase from *S. viridochromogenes* is known [29–30], but no enzyme for the biosynthesis of **12** has been reported before. The enzymes described here were named *Streptomyces lavendulae* (−)-Amorpha-4,11-diene Synthase (SlADS) and *Streptomyces subrutilus* (−)-Amorpha-4,11-diene Synthase (SsADS). These two enzymes belong to a clade of closely related enzymes with a pairwise identity of 70%, suggesting that (−)-amorpha-4,11-diene synthases also occur in several other streptomycetes (Figure S32, Supporting Information File 1).

**Figure 5:**
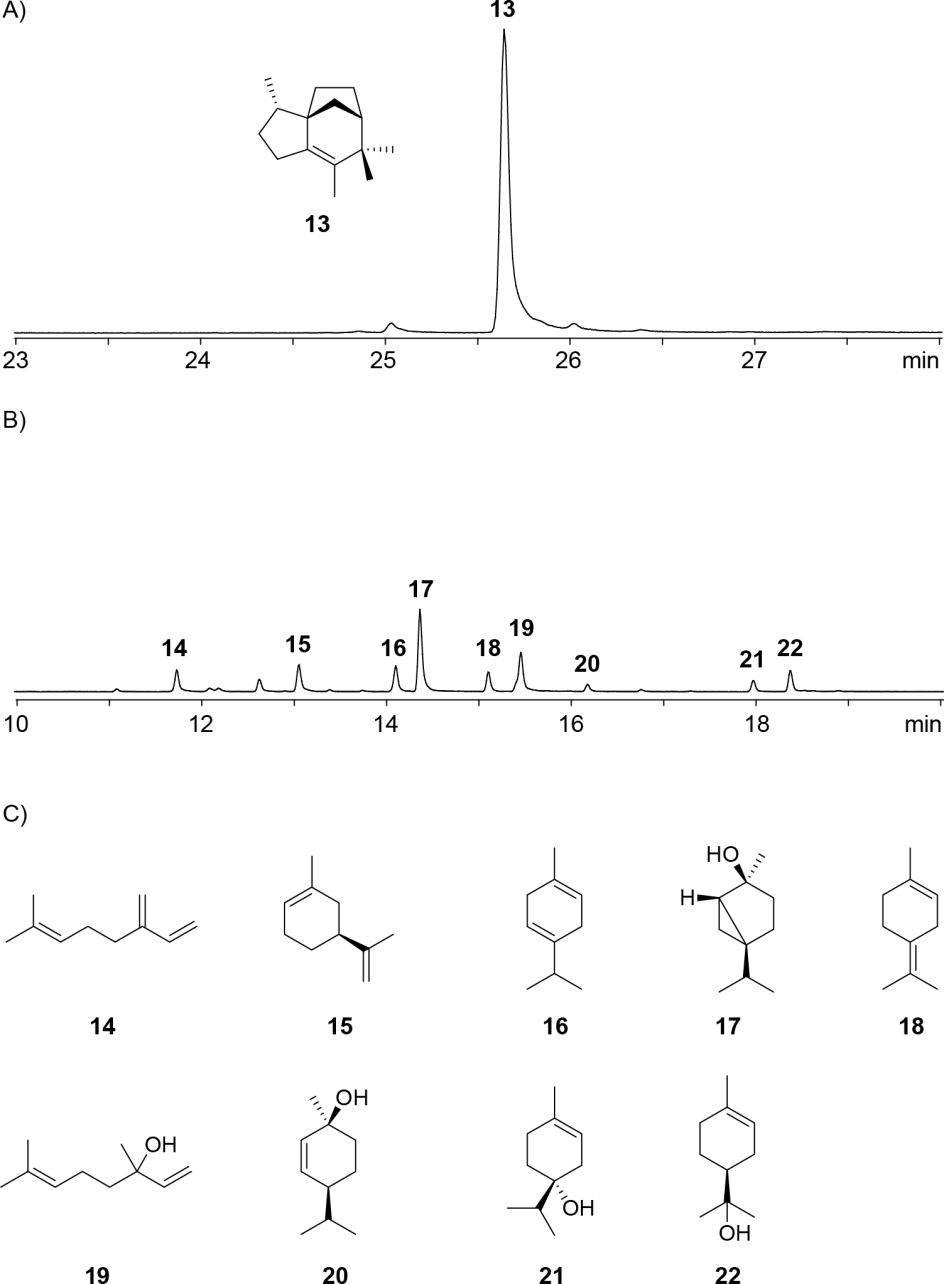
Total ion chromatograms of the products obtained with NbEIZS A) from FPP and B) from GPP, and C) structures of identified monoterpenes (only relative configurations are known). Peak numbers in B) refer to compound numbers in C).

The terpene synthase homolog from *N. brevicatena* (Table 1, entry 5) showed the highly conserved motifs with a modified aspartate-rich region (^86^DDHRN) and the NSE triad ^227^NDLHSMPKE (Figure S33, Supporting Information File 1). This enzyme is closely related to the *epi*-isozizaene synthase from *S. coelicolor* (EIZS) [24], but is with an amino acid identity of only 48% sufficiently distant so that another function could be expected (Figure S34, Supporting Information File 1). However, the incubation with FPP resulted in the efficient formation of *epi*-isozizaene (**13**) as a single product (Figure 5), confirming the same function as for known EIZS and identifying the investigated enzyme as *Nocardia brevicatena* *epi*-isozizaene synthase (NbEIZS). GGPP was not converted, but the incubation with GPP resulted in the production of a complex mixture of monoterpenes including myrcene (**14**), sylvestrene (**15**), γ-terpinene (**16**), *cis*-sabinene hydrate (**17**), terpinolene (**18**), linalool (**19**), *cis*-*p*-ment-2-en-1-ol (**20**), terpinen-4-ol (**21**) and α-terpineol (**22**). All these compounds were identified by comparison of their mass spectra to library spectra and of their gas chromatographic retention indices to literature data (Table S5, Supporting Information File 1). This result stands in contrast to the inability of *epi*-isozizaene synthase from *Streptomyces bungoensis* to accept GPP as a substrate [45].

**Figure 6:**
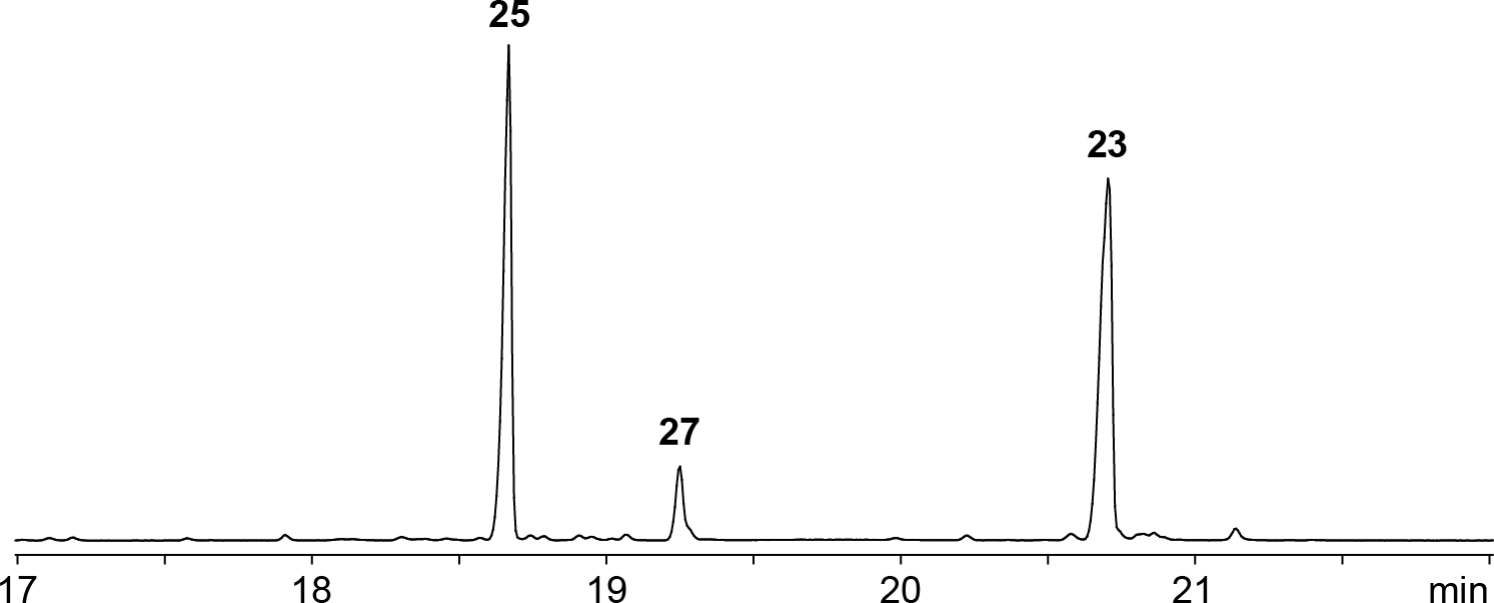
Total ion chromatogram of the products obtained with SfES. Peak numbers refer to compound numbers in Scheme 1.

**Scheme 1:**
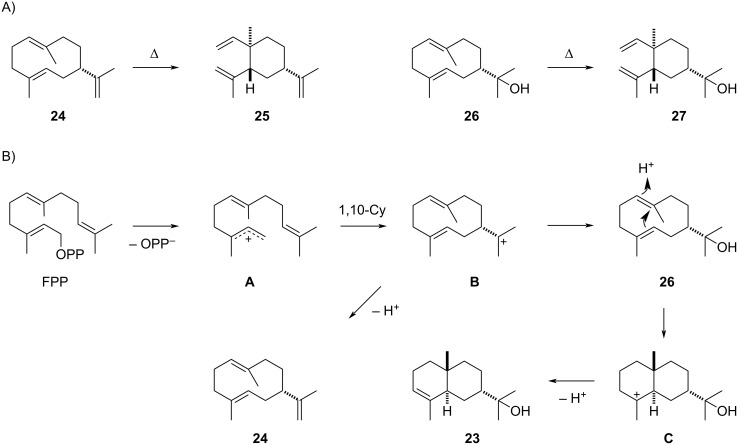
A) Cope rearrangement of **24** and **26**. B) Cyclisation mechanism from FPP to **23**, identifying compound **26** as a biosynthetic intermediate and **24** as a side product.

The terpene synthase homolog from *S. flavidovirens* (Table 1, entry 6) exhibited all highly conserved residues required for enzyme function including the aspartate-rich motif ^80^DDQFD and the NSE triad ^221^NDIHSFERE (Figure S35, Supporting Information File 1). This enzyme is with an identity of 78% closely related to the 7-*epi*-α-eudesmol synthase from *S. viridochromogenes* (SvES) [29–30], but forms a separate clade with nine other terpene synthase homologs (Figure S36, Supporting Information File 1), suggesting that it could have a different function. The incubation with FPP yielded 7-*epi*-α-eudesmol (**23**) as the main product, besides germacrene A (**24**) and hedycaryol (**26**) that were detected by their Cope rearrangement products elemene (**25**) and elemol (**27**) formed during GC–MS analysis (Figure 6 and Scheme 1A). These compounds are also observed with 7-*epi*-α-eudesmol synthase from *S. viridochromogenes*, demonstrating that the phylogenetic distance of the enzyme from *S. flavidovirens* is not associated with a different enzyme function. The conversion of GPP gave only trace amounts of acyclic products (geraniol and linalool), while GGPP and GFPP were not accepted as substrate. Taken together, the newly characterised enzyme has a main activity for the formation of **23** and can thus be described as *Streptomyces flavidovirens* 7-*epi*-α-Eudesmol Synthase (SfES).

The formation of **24** and **26** can be well understood from the cyclisation mechanism towards **23** (Scheme 1B). After substrate ionisation to **A** a 1,10-cyclisation leads to the (*E*,*E*)-germacradienyl cation (**B**) that can either be deprotonated to **24** or captured with water to yield **26**. Both compounds are important neutral intermediates in sesquiterpene biosynthesis that can be reactivated by reprotonation for a second cyclisation to eudesmane (6,6-bicyclic) or guaiane (7,5-bicyclic) sesquiterpene hydrocarbons or alcohols, respectively [46–47]. Starting from **26**, such a protonation induced cyclisation can lead to **C** that is the direct precursor of **23** by deprotonation.

**Figure 7:**
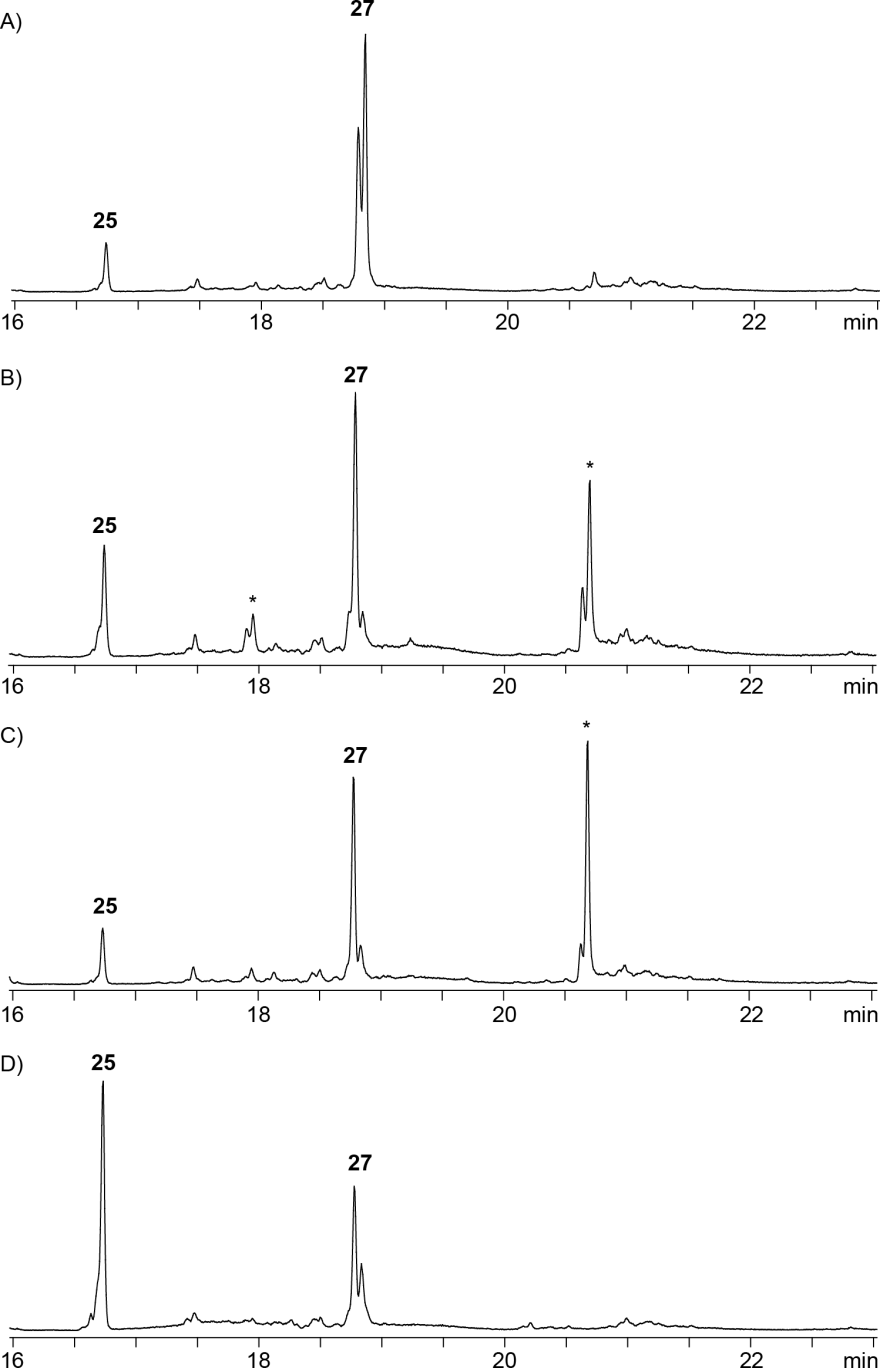
Total ion chromatograms of the products obtained with A) SsHS, B) ScHS, C) SfHS, and D) SmGAS. Peak numbers refer to compound numbers in Scheme 1. Asterisks indicate acyclic products and contaminants such as plasticisers.

Furthermore, four closely related terpene synthase homologs from one clade in the phylogenetic tree were investigated (Figure S37, Supporting Information File 1), including enzymes from *S. sclerotialus*, *S. catenulae*, *S. ficellus* and *S. morookaense* (Table 1, entries 7–10). These enzymes showed a pairwise identity of 63% and all exhibited the highly conserved motifs of type I terpene synthases (Figures S38–S41, Supporting Information File 1), only for the enzyme from *S. sclerotialus* the pyrophosphate sensor is missing (Figure S38, Supporting Information File 1) and for the enzyme from *S. catenulae* the RY pair is modified to RF (Figure S39, Supporting Information File 1). The closest characterised homolog of these enzymes is the spiroviolene synthase from *S. violens* [27] with amino acid sequence identities between 32% and 36%. All four enzymes did not accept GPP, GGPP or GFPP, but converted FPP with low product formation into varying mixtures of hedycaryol and germacrene A, detected as Cope rearrangement products **25** and **27**, eventually besides acyclic products (Figure 7). According to the source organism, the enzymes were named as hedycaryol synthases (HS) SsHS, ScHS and SfHS, and the enzyme from *S. morookaense* with **24** as main product was named *Streptomyces morookaense* Germacrene A Synthase (SmGAS). Notably, these enzymes are unrelated to the previously characterised hedycaryol synthase from *Kitasatospora setae* [48] and the germacrene A synthase from *M. marina* [32]. The low productivity of the four enzymes together with the low sequence conservation between them and the deviations in their conserved motifs may point to a pseudogenisation within this branch of the phylogenetic tree. This view is further supported by the observation that three more genes from the same branch from *S. subrutilus*, *S. natalensis* and *S. violens* were in one case not expressed and in two cases only yielded soluble, but inactive enzymes with any of the tested substrates GPP, FPP, GGPP and GFPP (Table 1, entries 11–13).

### Diterpene and sesterterpene synthases

**Figure 8:**
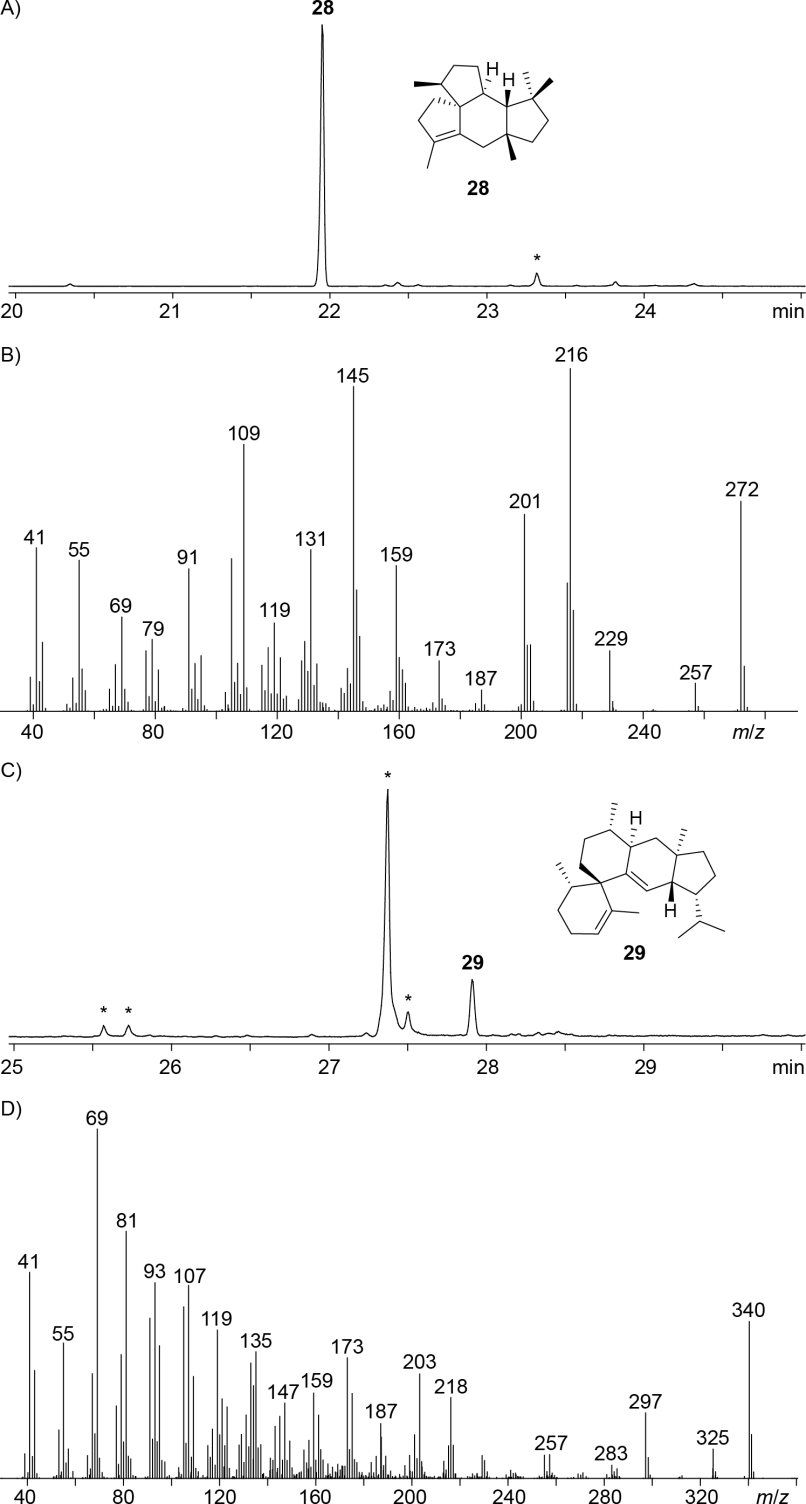
A) Total ion chromatogram of the products obtained from GGPP with KkAS, B) EI mass spectrum of allokutznerene (**28**), C) total ion chromatogram of the products obtained from GFPP with sesterviolene synthase from *Streptomyces* sp. Tü 2975, and D) EI mass spectrum of sesterviolene (**29**). Asterisks indicate acyclic products and contaminants such as plasticisers.

One more terpene synthase homolog from *K. kofuensis* (Table 1, entry 14) revealed all highly conserved motifs (Figure S42, Supporting Information File 1) with the aspartate-rich region ^81^DDINCD and a slightly modified NSE triad (^224^DDLFSYGKE). This enzyme is most closely related to the cattleyene synthase (CyS) from *Streptomyces cattleya* that shows the same sequence deviation in the NSE triad and has 58% identity [49], and to phomopsene synthase from *A. albata* with 36% identity (Figure S43, Supporting Information File 1) [50]. The incubation of GPP, FPP and GFPP with the purified protein only resulted in acyclic products, while with GGPP an efficient conversion with high selectivity into allokutznerene (**28**), known as a minor product of bacterial phomopsene synthase [50], was observed (Figure 8A and 8B). It is interesting to note that the low sequence identity between phomopsene synthase and *Kutzneria konfuensis* Allokutznerene Synthase (KkAS) still leads to the same product. The phylogenetic tree in Figure S43 (Supporting Information File 1) implies that three more enzymes from the genus *Kutzneria* may act as allokutznerene synthases. While also a fungal phomopsene synthase is known from *Phomopsis amygdali* [51], the biosynthesis of **28** is so far limited to bacteria.

Finally, a terpene synthase homolog from *Streptomyces* sp. Tü 2975 was investigated in this study (Table 1, entry 15). The enzyme contains all conserved sequences with a slightly modified aspartate-rich motif (^88^DDFIV) and the NSE triad ^227^NDRYSFCKE, and is with an amino acid sequence identity of 85% closely related to the recently reported sesterviolene synthase from *Streptomyces violarus* (SvSS) [52]. Accordingly, also the enzyme from *Streptomyces* sp. Tü 2975 catalysed the conversion of GFPP into sesterviolene (**29**, Figure 8C and 8D), but GPP, FPP and GGPP were not taken as substrate. The newly identified enzyme was designated *Streptomyces* sp. Tü 2975 Sesterviolene Synthase (StSS).

## Conclusion

Despite the accumulated knowledge on bacterial terpene synthases, the scattered distribution of sesqui-, di- and sesterterpene synthases in the phylogenetic tree of Figure 2 demonstrates that it is not possible to predict the substrate chain length of bacterial terpene synthases from a phylogenetic analysis. However, the phylogeny driven investigation of bacterial type I terpene synthase homologs can give access to terpene synthases with novel functions with a good success rate. This approach resulted in the identification of the first sesquiterpene synthases for (+)-δ-cadinol and (+)-α-cadinene, in addition to the first bacterial (−)-amorpha-4,11-diene synthase. This enzyme function was previously only known from *Artemisia annua* in which the (−)-amorpha-4,11-diene synthase is involved in the biosynthesis of artemisinin. The newly discovered bacterial enzyme may be useful for future heterologous pathway reconstitution towards this important drug [52–54]. Enzymes rather closely related to known *epi*-isozizaene [24] and 7-*epi*-α-eudesmol synthases [29–30], but sufficiently distant to expect novel functions, were shown to still form the same products as the previously characterised enzymes. However, the *epi*-isozizaene synthase from *Nocardia brevifolia* exhibited in contrast to the known enzyme from *Streptomyces bungoensis* [45] a substantial monoterpene synthase activity with formation of a product mixture. The 7-*epi*-α-eudesmol synthase from *Streptomyces flavidovirens* showed a loss of function and selectivity with formation of hedycaryol and germacrene A. This observation may be interpreted as the starting point towards pseudogenisation within the branch of 7-*epi*-α-eudesmol synthases. Pseudogenisation may be more advanced within a previously uninvestigated clade of terpene synthase homologs that is distant to other characterised enzymes. Within this clade not only four enzymes producing mixtures of hedycaryol and germacrene A were identified, but also two inactive enzymes were obtained and one enzyme was not expressed. Another interesting discovery was the identification of a diterpene synthase from *Kutzneria kofuensis* that selectively produces allokutznerene. This compound was previously only known as a side product from a closely related phomopsene synthase from *Allokutzneria albata*. The availability of a selective enzyme for allokutznerene is particularly interesting, because the separation of phomopsene and allokutznerene is reportedly very difficult [50]. Last but not least, a sesterterpene synthase for sesterviolene was discovered from *Streptomyces* sp. Tü 2975 that is closely related to the known enzyme from *Streptomyces violarus* [15].

The description of terpene synthases with novel functions as reported in this study is not only important for specific potential applications such as the usage of the bacterial (−)-amorpha-4,11-diene synthase for a pathway reconstruction towards artemisinin. The increased knowledge about terpene synthases together with the structures of their products will also be of interest for machine learning approaches to enable the prediction of terpene synthase functions from their amino acid sequences. Both aspects are relevant arguments to continue the research on terpene synthases, despite the fact that already many enzymes of this class have been described.

## Supporting Information

File 1 Additional information and spectra.
