# Supplementary material for: Functional characterisation of twelve terpene synthases from actinobacteria
Source: Beilstein J Org Chem. 2023 Sep 15;19:1386–98. doi: 10.3762/bjoc.19.100 (PMC10509563; doi:10.3762/bjoc.19.100)
Supplement: File 1 — Additional information and spectra. [file Beilstein_J_Org_Chem-19-1386-s001.pdf]

BEILSTEIN JOURNAL OF ORGANIC CHEMISTRY

Supporting Information

for

Functional characterisation of twelve terpene synthases from actinobacteria

Anuj K. Chhalodia, Houchao Xu, Georges B. Tabekoueng, Binbin Gu,
Kizerbo A. Taizoumbe, Lukas Lauterbach and Jeroen S. Dickschat

Beilstein J. Org. Chem. **2023,** *19,* 1386–1398. doi:10.3762/bjoc.19.100

Additional information and spectra

License and Terms: This is a supporting information file under the terms of the Creative Commons Attribution License (https://creativecommons.org/licenses/by/4.0). Please note that the reuse, redistribution and reproduction in particular requires that the author(s) and source are credited and that individual graphics may be subject to special legal provisions.
The license is subject to the *Beilstein Journal of Organic Chemistry* terms and conditions: (https://www.beilstein-journals.org/bjoc/terms)

Construction of phylogenetic tree

The amino acid sequences of 4018 bacterial terpene synthase homologs were obtained through a BLAST search in genome sequenced bacteria and used to construct the phylogenetic tree (Figure 2 of main text). All of the included sequences were individually inspected for the presence of the highly conserved motifs required for type I terpene synthase activity (i.e., the aspartate-rich motif, the NSE triad, the pyrophosphate sensor and the RY pair). The tree was constructed through the tree builder function of Geneious (alignment type: global alignment with free end gaps, cost matrix: Blosum45, genetic distance model: Jukes-Cantor, tree build method: neighbor-joining, gap open panelty: 8, gap extension penalty: 2).

Strains and culture collections

Kutzneria kofuensis DSM 43851, *Streptomyces jumonjinensis* NRRL 5741 (= DSM 747), *Streptomyces lavendulae* NRRL B-2774 (= DSM 40069), *Streptomyces subrutilus* ATCC 27467 (= DSM 40445), *Streptomyces flavidovirens* DSM 40150, *Streptomyces sclerotialus* NRRL ISP-5269 (= DSM 43032), *Streptomyces catenulae* NRRL B-2342 (= DSM 40258), *Streptomyces ficellus* NRRL 8067 (= DSM 930), *Streptomyces morookaense* DSM 40503, *Streptomyces natalensis* NRRL B-5314 (= DSM 40357), *Streptomyces violens* NRRL ISP-5597 (= DSM 40597), and *Nocardia brevicatena* NBRC12119 (= DSM 43024) were obtained from the German Collection of Microorganisms and Cell Cultures (DSMZ). *Streptomyces* sp. Tü 2975 was obtained from Prof. Heike Brötz-Oesterhelt (University of Tübingen). All strains were grown in medium 65 medium at 28 °C, except *Nocardia brevicatena* NBRC12119 that was grown in medium 82 at 37 °C.

Gene cloning

gDNA was isolated from each of the actinomycetes using the phenol/chloroform method.[1] The genes encoding the enzymes as listed in Table 1 of the main text were amplified from gDNA by using PCR Q5 High-Fidelity DNA Polymerase (New England Biolabs) and the primers pairs listed in Table S1. These primers contain the priming sequences (in regular) and homology arms (in bold underlined) that have homologous sequences to the ends of the linearised pYE-Express vector (EcoRI and HindIII

digestion) for homologous recombination in yeast through the PEG/LiOAc method.[2,3] *Saccharomyces cerevisiae* FY834 was cultured for 3 days in YPAD medium and the plasmids containing the integrated genes were isolated with the Zymoprep Yeast Plasmid Miniprep II kit (Zymoresearch, Irvine, CA, USA), followed by the introduction of the plasmids into *E. coli* BL21 (DE3) through electroporation. The transformants were grown on LB agar plates amended with kanamycin (50 μg/mL) at 37 °C overnight. Single colonies were selected and cultured in LB medium (10 mL) with kanamycin (50 μg/mL) at 37 °C overnight for the isolation of plasmid DNA by using the PureYield Plasmid Miniprep System (Promega, Madison, WI, USA). The sequences of the cloned genes were verified by DNA sequencing.

Table S1. Primers used for gene cloning.

Primer[a]	Sequence[b]
MBB5895433_Fw	**GGCAGCCATATGGCTAGCATGACTGGTGGA**GTGACCATCTGCGACACAA
MBB5895433_Rv	**TCTCAGTGGTGGTGGTGGTGGTGCTCGAGTG**TCACGCCGCTGCGGGCT
WP_153520876_Fw	**GGCAGCCATATGGCTAGCATGACTGGTGGA**ATGGCCCCGCCCTTGAG
WP_153520876_Rv	**TCTCAGTGGTGGTGGTGGTGGTGCTCGAGTG**TCAGCTCTGGACGGGTGTCTGT
WP_078950427_Fw	**GGCAGCCATATGGCTAGCATGACTGGTGG**AATGACCCAGCTCAACTCTTT
WP_078950427_Rv	**TCTCAGTGGTGGTGGTGGTGGTGCTCGAG**TGTCATGCCACCGAAACCAGGC
WP_150516140_Fw	**GGCAGCCATATGGCTAGCATGACTGGTGGA**ATGACCCAGCTCAACTCTTTCTCT GTTCC
WP_150516140_Rv	**TCTCAGTGGTGGTGGTGGTGGTGCTCGAGTG**TCATGCCACCCAGACCAGGTCC
WP_086008896_Fw	**GGCAGCCATATGGCTAGCATGACTGGTGGA**GTGACCGCGGTCTTCGAG
WP_086008896_Rv	**TCTCAGTGGTGGTGGTGGTGGTGCTCGAGT**TCACTGTTCTCGACCTTCTTCCAC
WP_028812116_Fw	**GGCAGCCATATGGCTAGCATGACTGGTG**GAGTGCCGCAGGACGTCCGTTTC
WP_028812116_Rv	**CTCAGTGGTGGTGGTGGTGGTGCTCGAGT**GTCAGACCGTGAGCAGGTCGTC
WP_030615021_Fw	**GGCAGCCATATGGCTAGCATGACTGGTGGA**ATGGCCACGATAACCGACCGCG
WP_030615021_Rv	**TCTCAGTGGTGGTGGTGGTGGTGCTCGAGTG**TTAGTCGCGCAGCTGGTCCCAC C
WP_051739595_Fw	**GGCAGCCATATGGCTAGCATGACTGGTGGA**ATGACCGTCACCCCCGACGTCC
WP_051739595_Rv	**TCTCAGTGGTGGTGGTGGTGGTGCTCGAGTG**TTAGGGAGCGTCGGGGGACGTC AGC
WP_156694351_Fw	**GGCAGCCATATGGCTAGCATGACTGGTGGA**ATGCCACCCTCCTACGCCGAGG
WP_156694351_Rv	**TCTCAGTGGTGGTGGTGGTGGTGCTCGAGTG**TTAGCCGTCGAGCTGCCGCCAC

WP_171082395_Fw	**GGCAGCCATATGGCTAGCATGACTGGTGGA**ATGACGGACACCTCGACGCG
WP_171082395_Rv	**TCTCAGTGGTGGTGGTGGTGGTGCTCGAGTG**TTACGCGGTGGCTGCGCAGG
WP_150522245_Fw	**GGCAGCCATATGGCTAGCATGACTGGTGGA**ATGCCGGCGTTCTACTGCCC
WP_150522245_Rv	**TCTCAGTGGTGGTGGTGGTGGTGCTCGAGTG**TTAGGCGCGGACGCCGACCTG
WP_037793252_Fw	**GGCAGCCATATGGCTAGCATGACTGGTGGA**ATGCCACCCGTCTACGCGGC
WP_037793252_Rv	**TCTCAGTGGTGGTGGTGGTGGTGCTCGAGTG**TTAGGCTCGCTCGGCAGCCCACC
WP_030249874_Fw	**GGCAGCCATATGGCTAGCATGACTGGTGGA**ATGCCGCCGTTCTACTGCCCCATC
WP_030249874_Rv	**TCTCAGTGGTGGTGGTGGTGGTGCTCGAGTG**TTAGCCGCGCAGCTGGTCCCAC
WP_184867163_Fw	**GGCAGCCATATGGCTAGCATGACTGGTGGA**ATGACGGACGACCAGCTGCT
WP_184867163_Rv	**TCTCAGTGGTGGTGGTGGTGGTGCTCGAGTG**CTAGAGTCCCAGCTCCGGGT
WP_159685978_Fw	**GGCAGCCATATGGCTAGCATGACTGGTGGA**ATGAACCTTGCCGACCTCCCCGAG
WP_159685978_Rv	**TCTCAGTGGTGGTGGTGGTGGTGCTCGAGTG**TCATGGCGTGATGTGCTCCCACCAC

[a] Primer names contain accession numbers to target genes. [b] Priming sequences are in regular and homology arms for homologous recombination in yeast are in bold and underlined.

Gene expression and protein purification

The *E. coli* BL21 (DE3) expression strains containing the respective plasmids were precultured in LB medium (10 mL) with kanamycin (50 μg/mL) overnight at 37 °C. The precultures were used to inoculate expression cultures in LB medium (8 L) containing kanamycin (50 μg/mL). The cultures were grown at 37 °C at 160 rpm until the OD_{600} = 0.5–0.6 was reached. The cultures were cooled to 18 °C and IPTG (0.4 mM in water, 1 mL L^{-1}) was added. The cultures were incubated for 18 h at 18 °C at 160 rpm. The cells were collected through centrifugation (3500 rpm, 1 h, 4 °C), resuspended in binding buffer (10 mL/L culture; 20 mM Na_2HPO_4, 500 mM NaCl, 20 mM imidazole, 1 mM $MgCl_2$, pH 7.4) and lysed by ultrasonication (10 × 1 min) on ice. The cell debris was removed by centrifugation (14600*g*, 7 min, 4 °C) and the soluble protein fraction was loaded onto a Ni^{2+}-NTA affinity chromatography column (Qiagen, Venlo, Netherlands) equilibrated with binding buffer. The column was washed with binding buffer (2 × 10 mL) and the desired protein was eluted with elution buffer (3 × 10 mL; 20 mM Na_2HPO_4, 500 mM NaCl, 500 mM imidazole, 1 mM $MgCl_2$, pH 7.4). The

concentration of enzymes was calculated by Bradford assay[4] and the purity of enzymes was checked by SDS-PAGE analysis. Protein yields were 1–5 mg mL^{-1}.

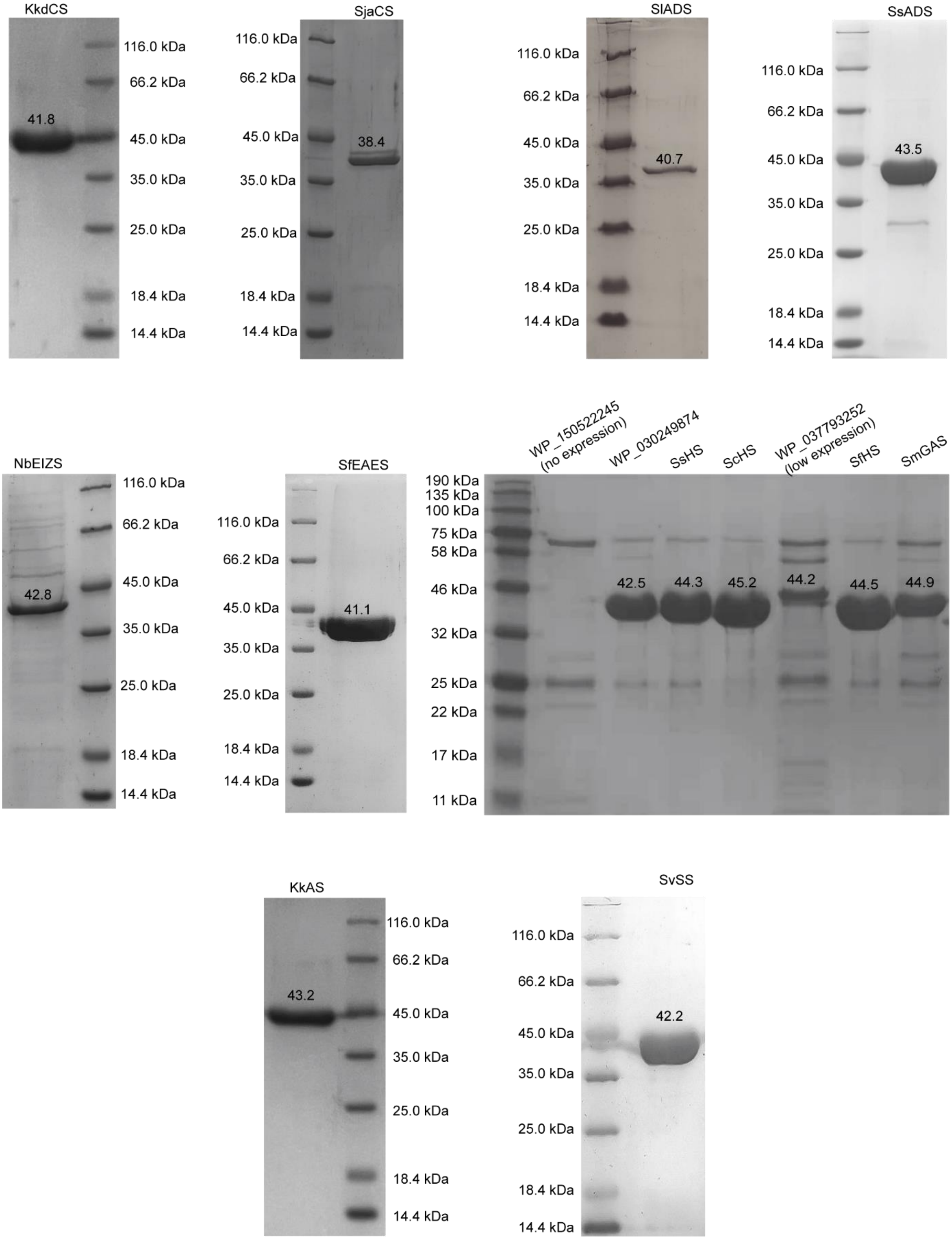

Figure S1. SDS-PAGE analysis of recombinant terpene synthases.

Incubation experiments and compound isolation

Test reactions were carried out with GPP, FPP, GGPP and GFPP to identify the substrate scope of recombinant enzymes. The substrate GPP, FPP, GGPP or GFPP (1 mg) was dissolved in substrate buffer (100 μL; 25 mM NH_4HCO_3). A protein preparation (final protein concentration: 0.3 mg mL^{-1}) and cyclodextrin (63 μL, 160 mM; final concentration: 10 mM) were added, and the sample was filled to 1 mL with incubation buffer (50 mM Tris/HCl, 10 mM $MgCl_2$, 20% glycerol, pH 8.2). The reaction mixture was incubated at 30 °C overnight. The product was extracted with hexane (100 μL), the extract was dried over $MgSO_4$ and analysed by GC–MS. Preperative scale reactions were performed by dissolving FPP triammonium salt (80 mg, 185 μmol) in substrate buffer (10 mL). A protein preparation obtained from 8 L expression culture was diluted with incubation buffer to adjust a protein concentration of 0.3 mg mL^{-1}. Cyclodextrin was added (63 μL/mL at a concentration of 160 mM, final concentration 10 mM). To the resulting mixture the FPP solution was slowly added dropwise and the reactions were incubated at 30 °C overnight. The mixture was extracted with pentane (3x 100 mL), the organic layers were collected, dried over $MgSO_4$, and the solvent was evaporated in vacuo. The product was purified by silica gel column chromatography.

GC–MS analyses

GC–MS analyses were carried out on a 7890B/5977A series gas chromatography/mass selective detector (Agilent, Santa Clara, CA, USA). The GC was equipped with an HP5-MS fused silica capillary column (30 m, 0.25 mm i. d., 0.50 µm film; Agilent) and operated using the settings: 1) inlet pressure: 77.1 kPa, He at 23.3 mL min^{-1}, 2) injection volume: 1 µL, 3) temperature program: 5 min at 50 °C, then increasing by 5 °C min^{-1} or 10 °C min^{-1} to 320 °C, 4) splitless or split ratio 50:1, 60 s valve time, and 5) carrier gas: He at 1 mL min^{-1}. The MS was operated with the same settings: 1) source: 230 °C, 2) transfer line: 250 °C, 3) quadrupole: 150 °C and 4) electron energy: 70 eV. Retention indices (I) were determined from a homologous series of *n*-alkanes (C_7–C_{40}).

NMR spectroscopy

NMR spectra were recorded on a Bruker (Billerica, MA, USA) Avance III HD Cryo (700 MHz) and AV III HD Prodigy (500 MHz) NMR spectrometer. Spectra were referenced

against solvent signals (^{1}H-NMR, residual proton signals: $CDCl_3$ δ = 7.26 ppm, C_6D_6 δ = 7.16, D_2O δ = 4.79; ^{13}C-NMR: $CDCl_3$ δ = 77.16 ppm, C_6D_6 δ = 128.06 ppm).[5]

MTICDTTLLWCPIPPGIHPNWRQWERDTVAWLESFALEDEQREKKRLQAIIAGELAGRTILS
CDDPPGAQFSTDSLMWLFAFDDAYCDEGRYSHDPAAMAMLVAEMGRIAETGRTVSTSPLARA
LAELRSRLDVLASPAQTARWVHAMKGYLGYQVWEAAFRHTGTIPTLDEYAVARIRNGSMEVC
AMTLDIAEGYEVPAAEIDRPDVRALTEMACSLVGWDNDIASYYKEHERSGDRINLVDVIADQ
KGSTPAEALPSAIALRDAVLARYLELRDEIEPHVGSLTWRYIGGLSAWIRGNLDWSANTARY
RRPDCPTVAVTSSREYATGDCPQPPGIAWWWTADPTQPAAA

Figure S2. Amino acid sequence of KkdCS (MBB5895433). Highly conserved residues are highlighted in yellow.

δ-Cadinol (10). This compound was isolated by column chromatography on silica gel with petrol ether / Et_2O (4:1). Yield: 5.5 mg, 25.0 μmol, 14%, from 80.0 mg (185 μmol) FPP triammonium salt. TLC (ether / Et_2O = 4:1): R_f = 0.3. GC (HP-5MS): *I* = 1665. Optical rotary power: $[\alpha]_D^{25}$ = +95.9 (*c* 0.55, CH_2Cl_2). The EI mass spectrum is shown in Figure 3B of main text and NMR data are given in Table S2.

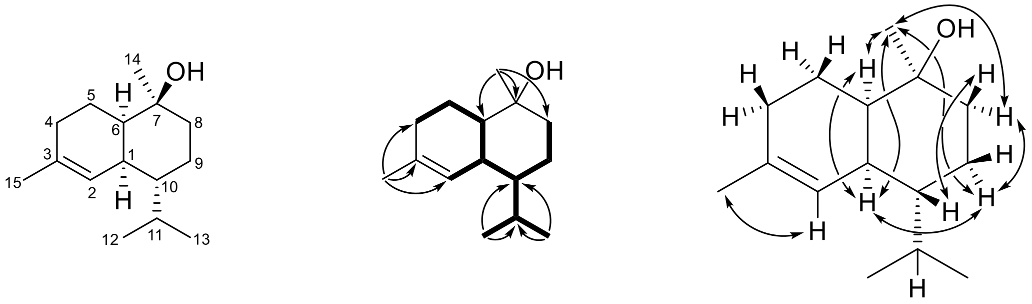

Figure S3. Structure elucidation of δ-cadinol (**10**). Bold: $^{1}H,^{1}H$-COSY, single headed arrows: key HMBC, and double headed arrows: NOESY correlations.

Table S2. NMR data of **10** in C_6D_6 recorded at 298 K.

		**10**	
C[a]	type	^{13}C[b]	^{1}H[b]
1	CH	37.18	1.97 (m)
2	CH	125.23	5.57 (dq, *J* = 5.5, 1.5).
3	Cq	134.32	–
4	CH_2	31.60	1.92 (m, 2H)
5	CH_2	18.89	2.00 (m)
			1.52 (m)
6	CH	46.04	1.54 (m)
7	Cq	71.67	–
8	CH_2	35.64	1.50 (m, H_β)
			1.36 (m, H_α)
9	CH_2	21.92	1.34 (m, H_β)
			0.96 (m, H_α)
10	CH	44.54	1.32 (m)
11	CH	26.80	2.00 (m)
12	CH_3	15.50	0.77 (d, *J* = 6.9, 3H)
13	CH_3	21.87	0.85 (d, *J* = 7.0, 3H)
14	CH_3	28.34	1.15 (s, 3H)
15	CH_3	23.86	1.63 (br s, 3H)

[a] Carbon numbering as shown in Figure S3. [b] Chemical shifts δ in ppm; multiplicity: s = singlet, d = doublet, q = quartet, m = multiplet, br = broad; coupling constants *J* are given in Hertz.

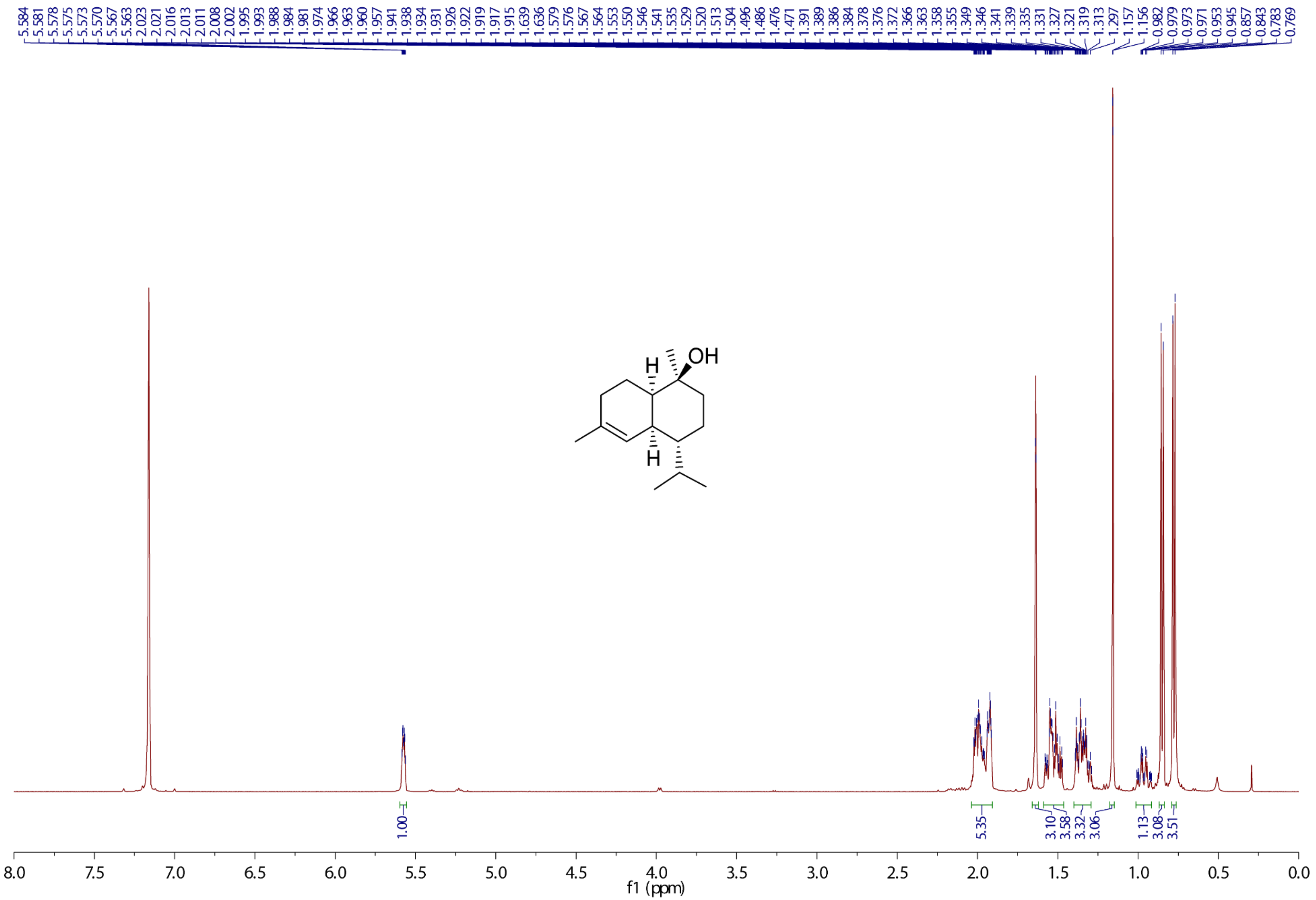

Figure S4. ^{1}H NMR spectrum of **10** (500 MHz, C_6D_6).

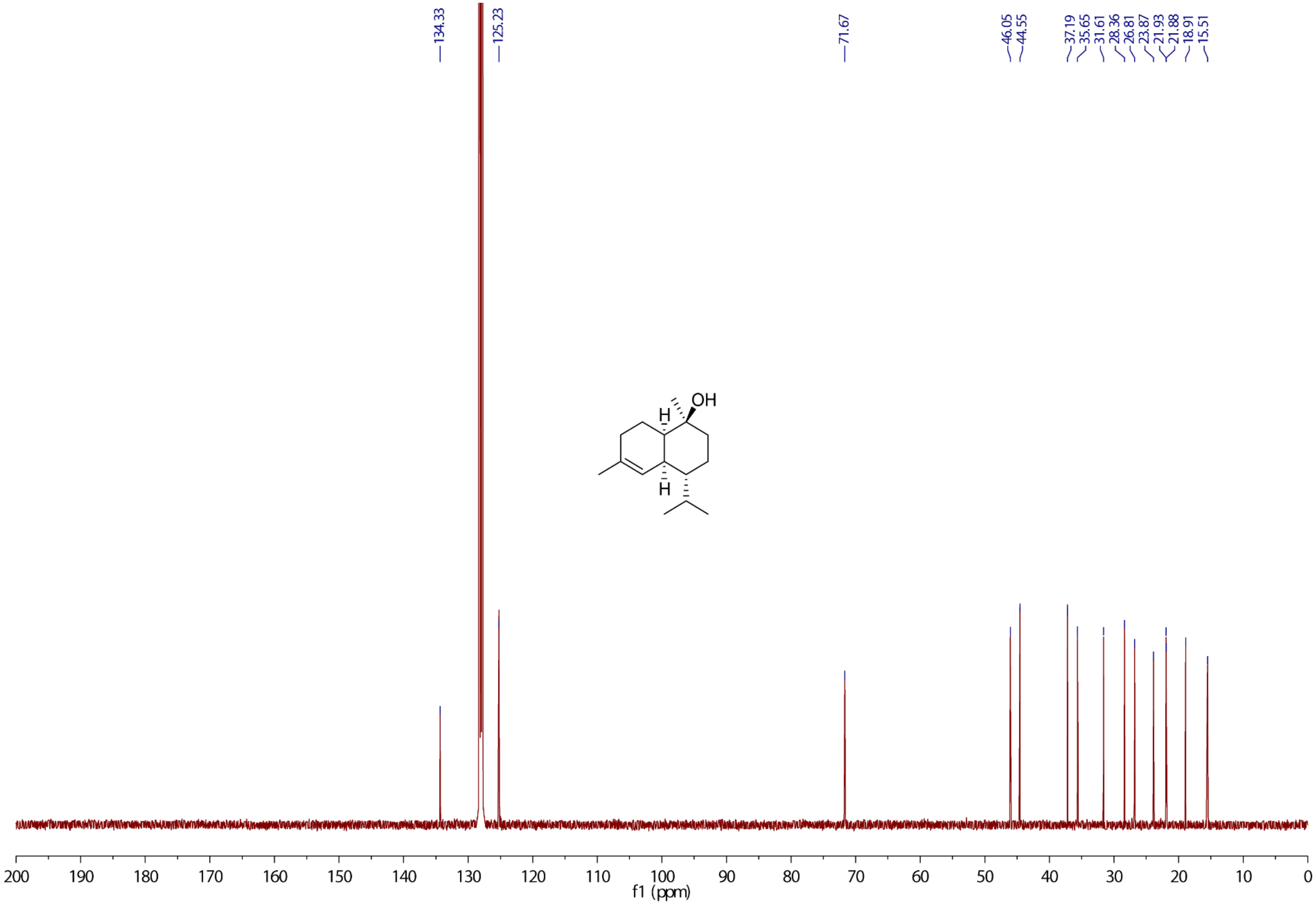

Figure S5. ^{13}C NMR spectrum of **10** (126 MHz, C_6D_6).

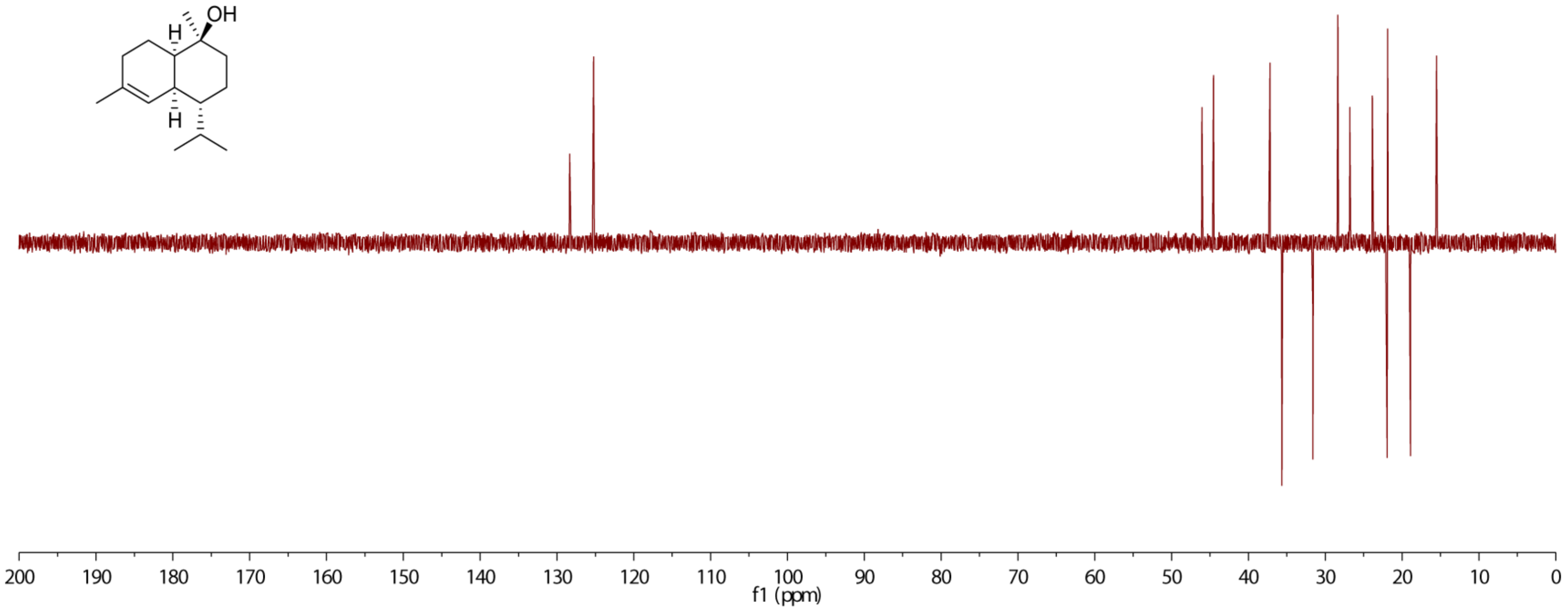

Figure S6. ^{13}C-DEPT135 spectrum of **10** (126 MHz, C_6D_6).

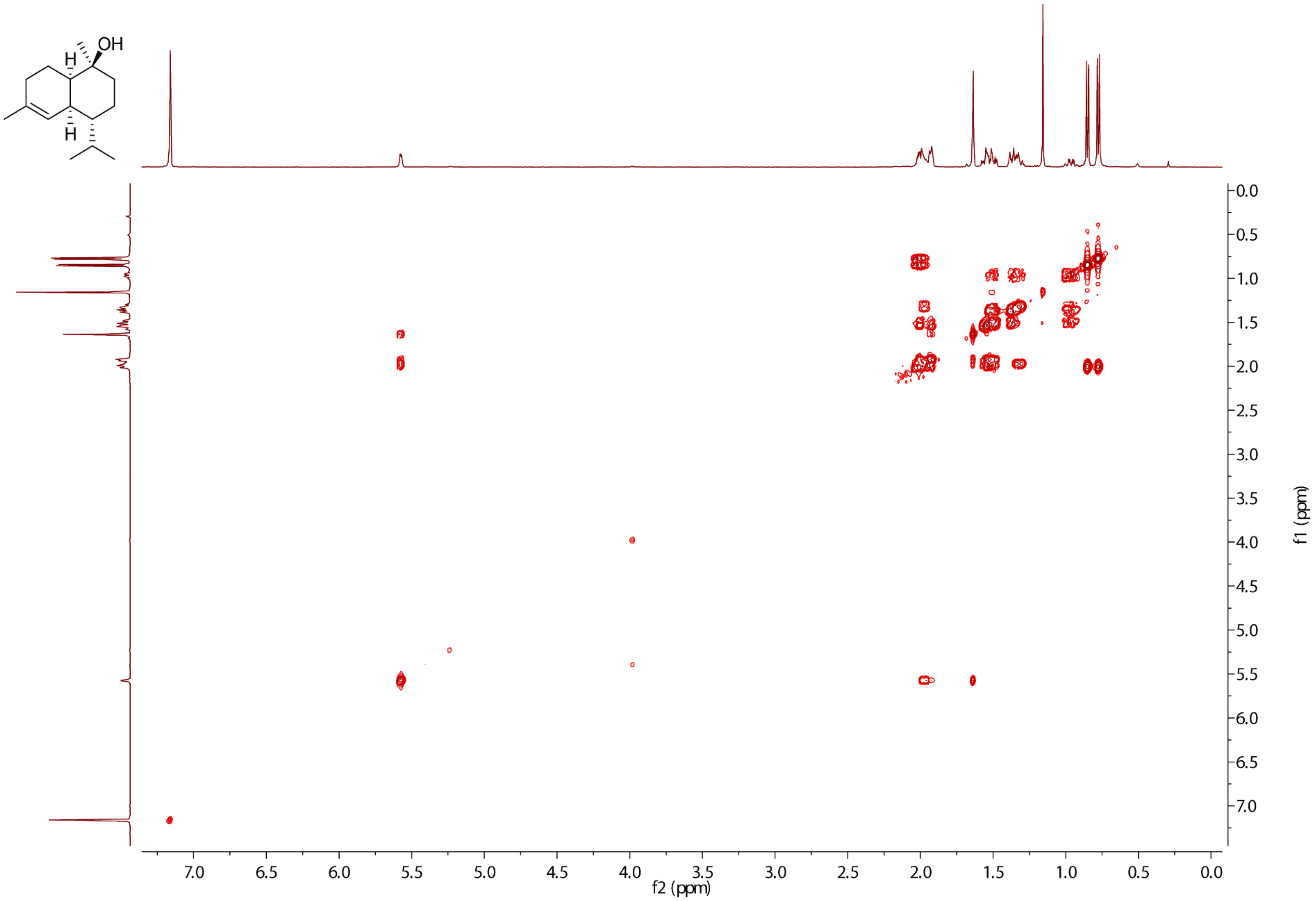

Figure S7. ^{1}H,^{1}H-COSY spectrum (C_6D_6) of **10**.

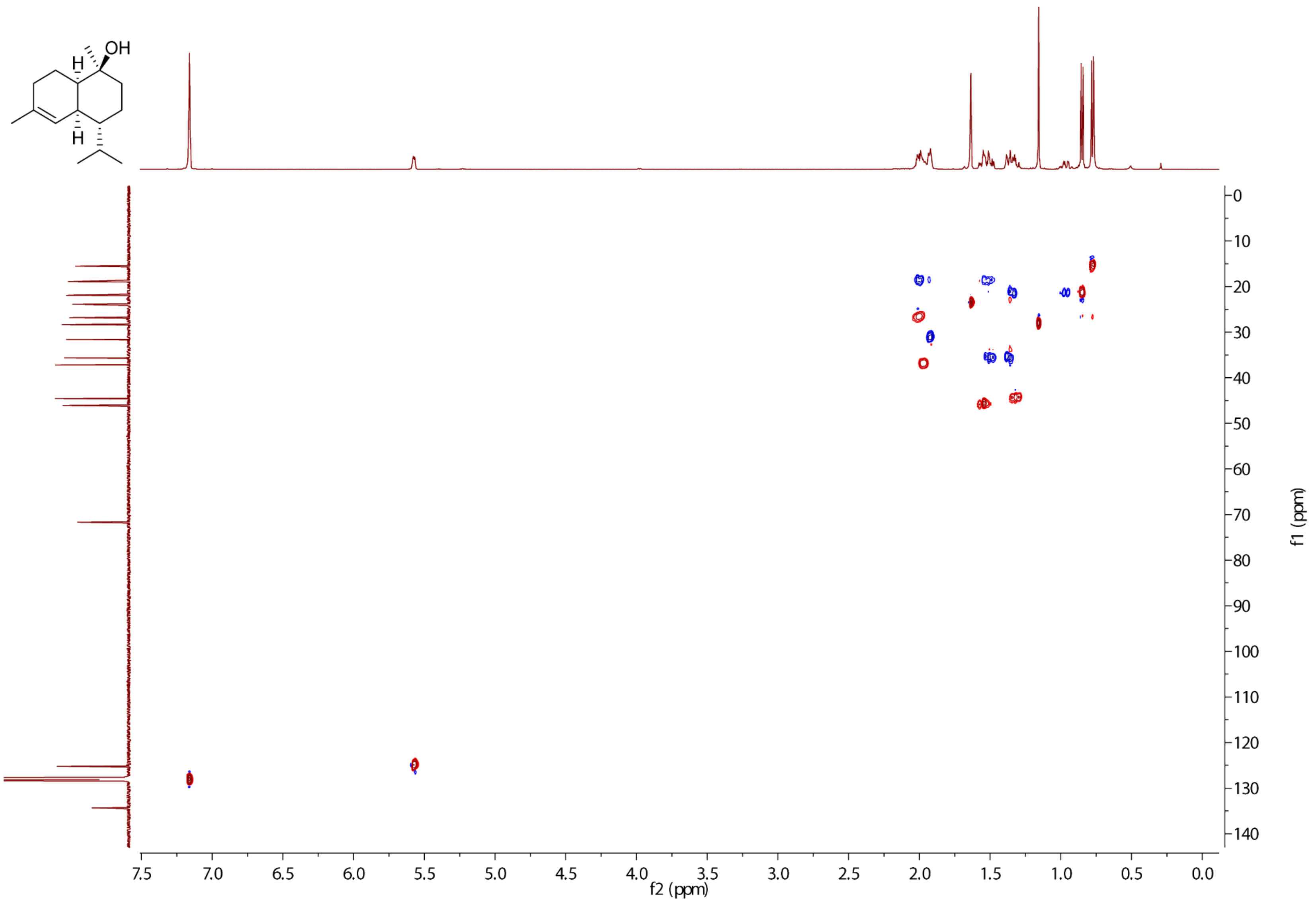

Figure S8. HSQC spectrum (C_6D_6) of **10**.

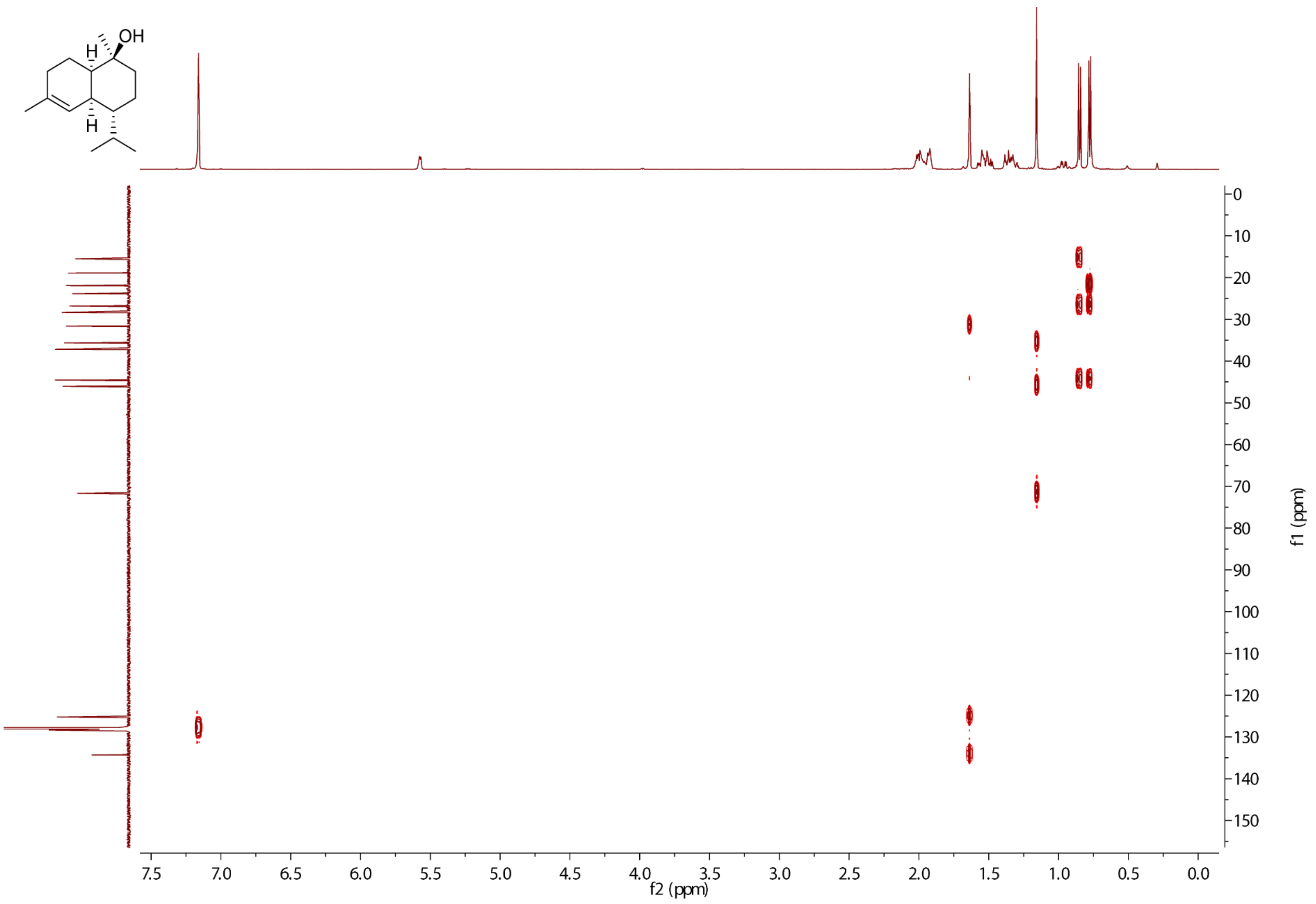

Figure S9. HMBC spectrum (C_6D_6) of **10**.

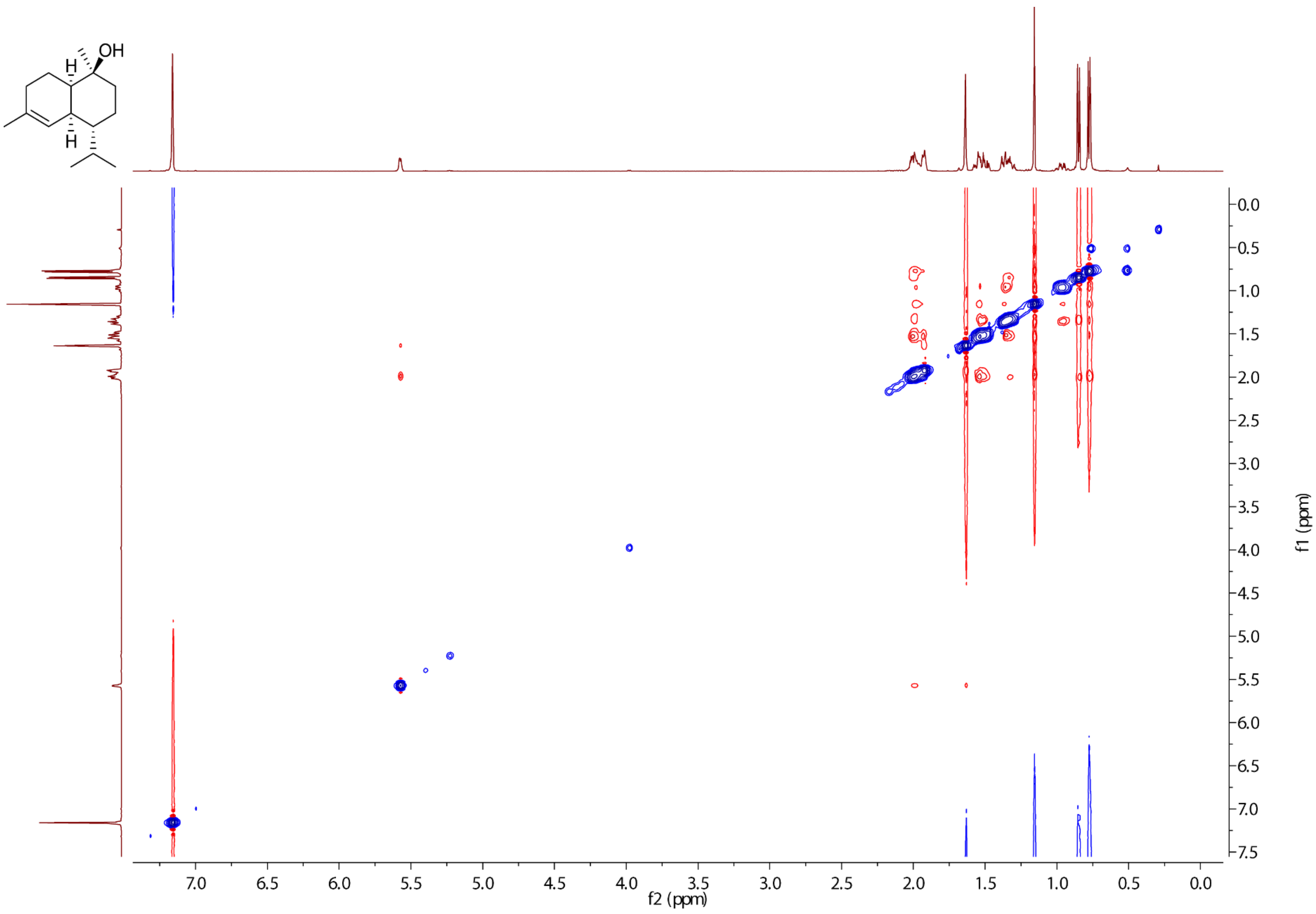

Figure S10. NOESY spectrum (C_6D_6) of **10**.

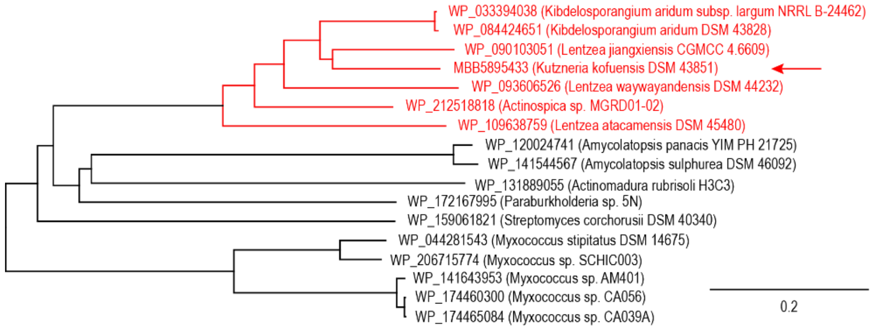

Figure S11. Detailed section of the phylogenetic tree shown in Figure 2 of main text. The red arrow indicates the characterised δ-cadinol synthase (KkdCS). The closely related enzymes shown in red share a pairwise identity of 69% and may have the same function.

MAPPLRIPPLHCPFPDDVHPEADTIDRTSLRWLDRFGLITDPATRARFGQSKIGWQASRTTP
HADAELVQLHSDWQMWLFAFDDVRSEESEAGGHPGRMARSLVPCLRILEDPDTPVRDEDPFT
AALRDLRRHLGLVAGPLQLDRFITSVLGYWFAQVWEAGNRADAVWPTVEEYTAMRVHTGAVP
TCLALIDVVGRFELPAAELARHEVKALTTKAVNVVCWANDIHSYEKEAARSSHPVNLPTLLH
RRDGGTVQAAIDLAARMHDDEVAAYVELRSRVTAGPELERYLDGLQSWMRGNLTWSLSTGRY
RQTPVQS

Figure S12. Amino acid sequence of SjaCS (WP_153520876). Highly conserved residues are highlighted in yellow.

α-Cadinene (11). This compound was isolated through silica gel column chromatography with pentane. Yield: 1.3 mg, 6.4 μmol, 3.5%, from 80 mg (185 μmol) FPP triammonium salt. TLC (pentane): R_f = 0.72. GC (HP-5MS): I = 1560. Optical rotary power: $[\alpha]_D^{25}$ = +60.0 (c 0.015, C_6H_6). The EI mass spectrum is shown in Figure 3D of main text and NMR data are given in Table S3.

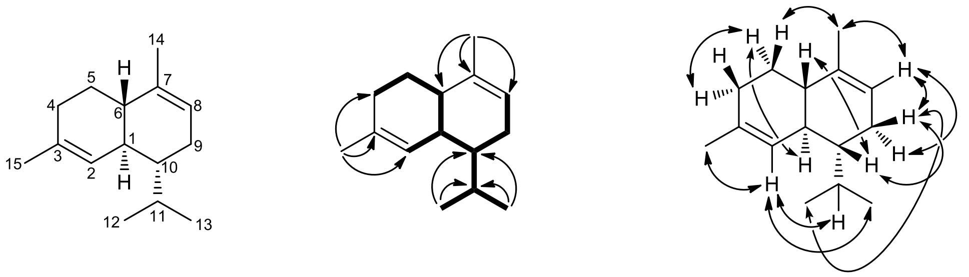

Figure S13. Structure elucidation of α-cadinene (**11**). Bold: ^{1}H,^{1}H-COSY, single headed arrows: key HMBC, and double headed arrows: NOESY correlations.

Table S3. NMR data of **11** in C_6D_6 recorded at 298 K.

		**11**	
C[a]	type	^{13}C[b]	^{1}H[b]
1	CH	41.57	1.97 (m, H$_\alpha$)
2	CH	122.90	5.72 (br s)
3	C$_q$	122.90	–
4	CH$_2$	31.64	1.97 (m, H$_\alpha$)
			1.91 (m, H$_\beta$)
5	CH$_2$	27.09	2.02 (m, H$_\beta$)
			1.25 (m, H$_\alpha$)
6	CH	43.05	1.94 (m, H$_\beta$)
7	C$_q$	135.71	–
8	CH	122.50	5.45 (br s)
9	CH$_2$	25.37	1.90 (m, H$_\beta$)
			1.83 (m, H$_\alpha$)
10	CH	42.79	1.51 (m, H$_\beta$)
11	CH	26.68	2.20 (m)
12	CH$_3$	14.72	0.80 (d, J = 6.9)
13	CH$_3$	21.12	0.87 (d, J = 7.0)
14	CH$_3$	21.00	1.69 (br s)
15	CH$_3$	23.99	1.67 (br s)

[a] Carbon numbering as shown in Figure S13. [b] Chemical shifts δ in ppm; multiplicity: s = singlet, d = doublet, m = multiplet, br = broad; coupling constants *J* are given in Hertz.

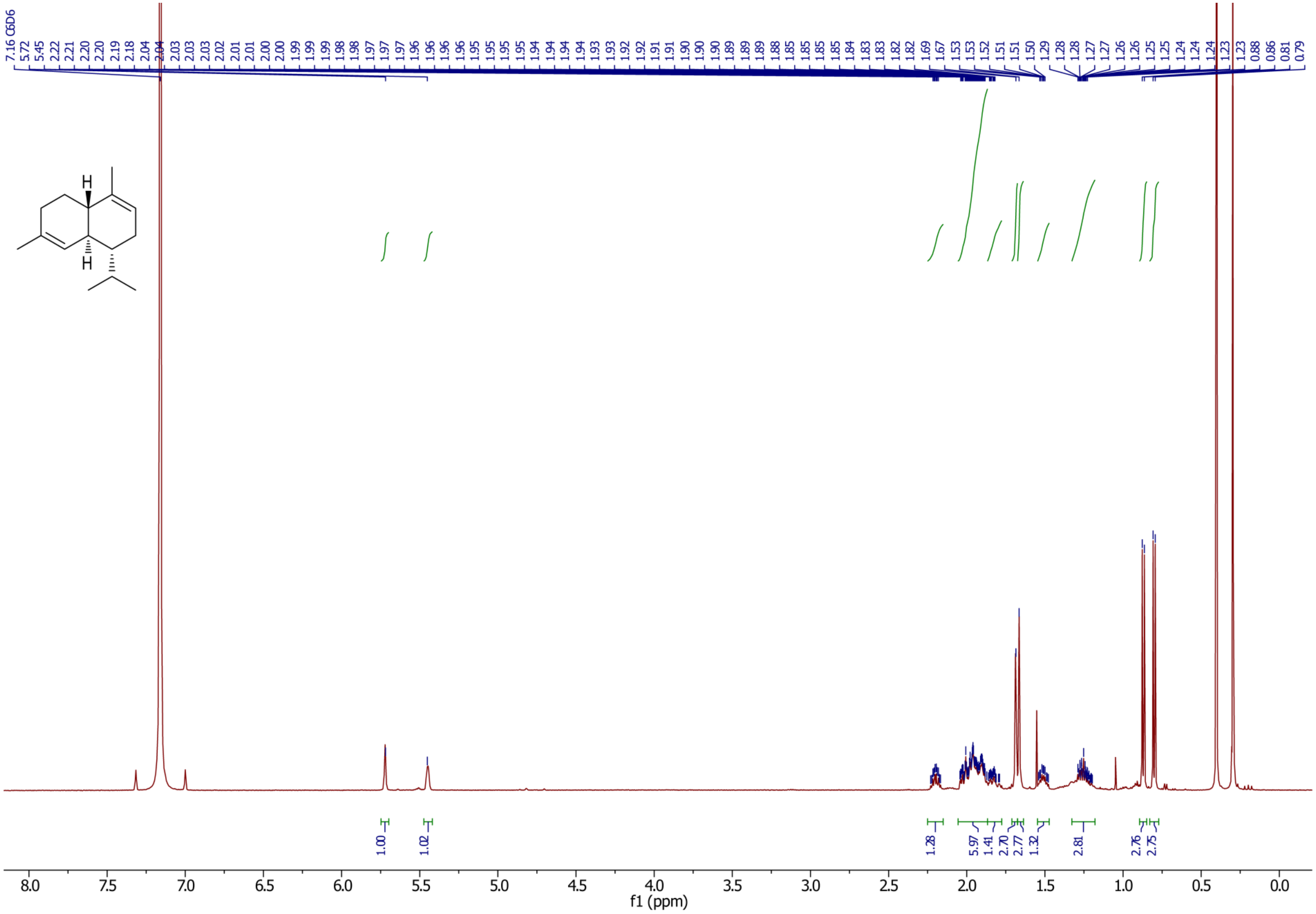

Figure S14. ^{1}H NMR spectrum of **11** (500 MHz, C_6D_6).

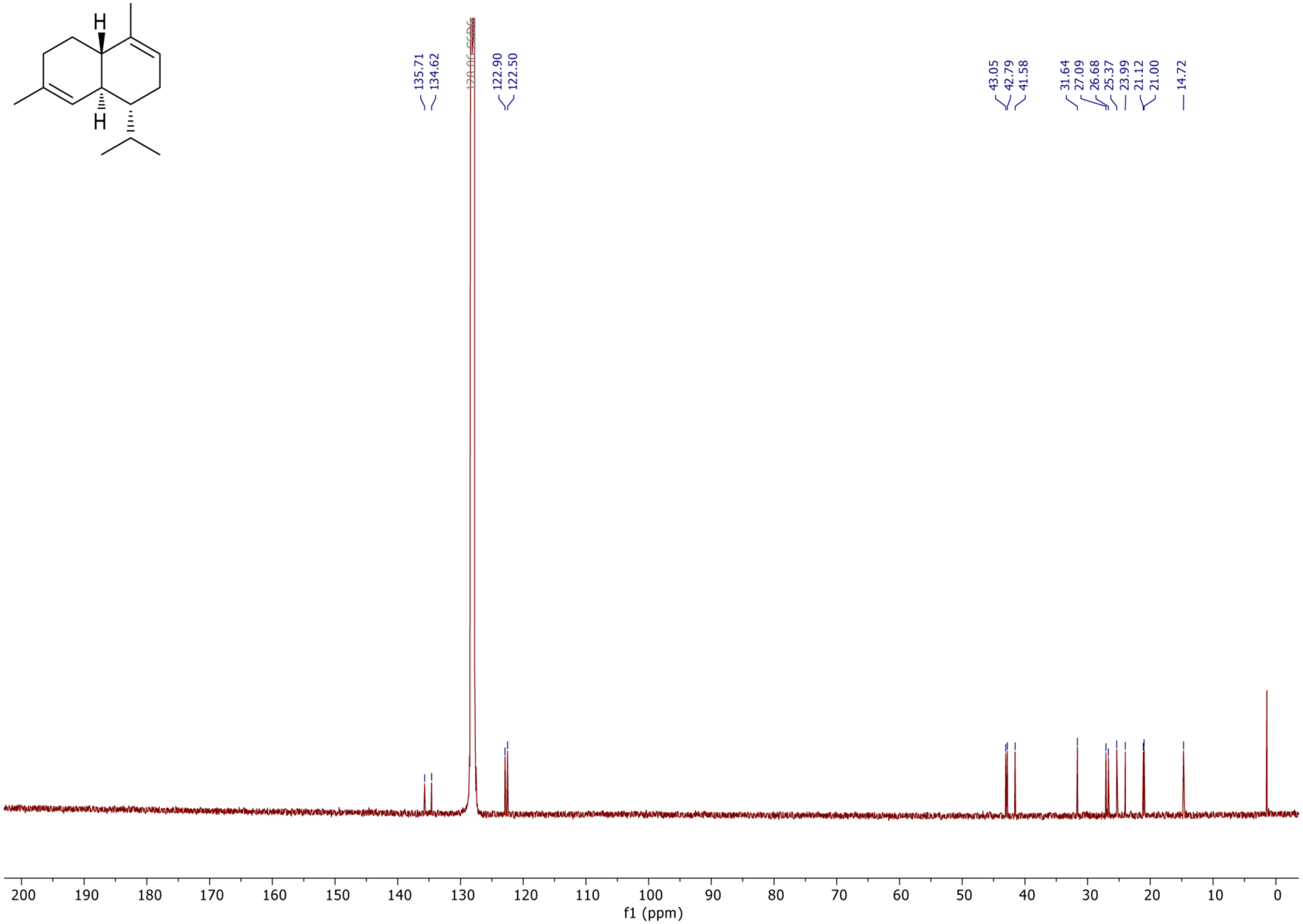

Figure S15. ^{13}C NMR spectrum of **11** (125 MHz, C_6D_6).

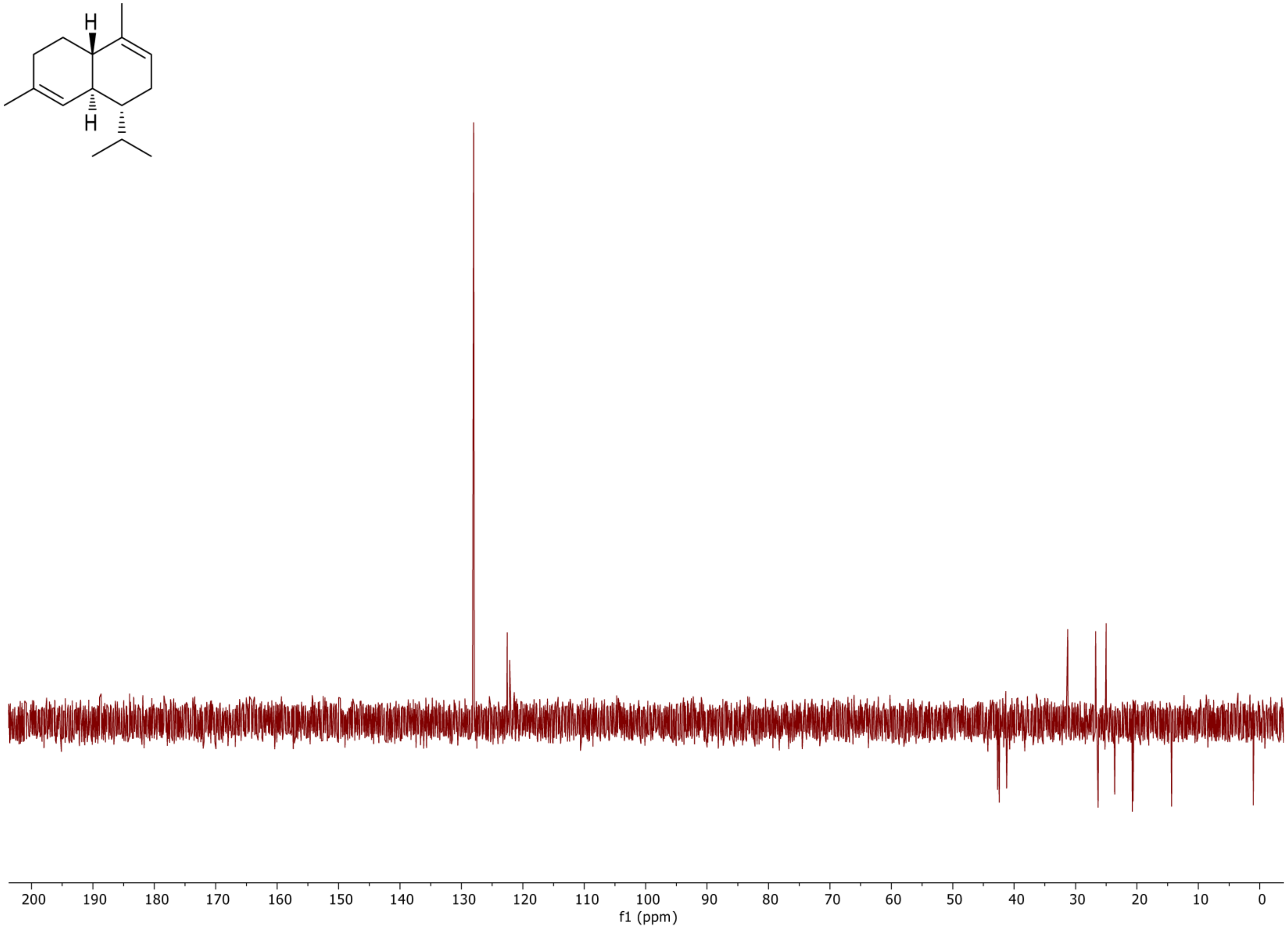

Figure S16. ^{13}C-DEPT135 spectrum of **11** (125 MHz, C_6D_6).

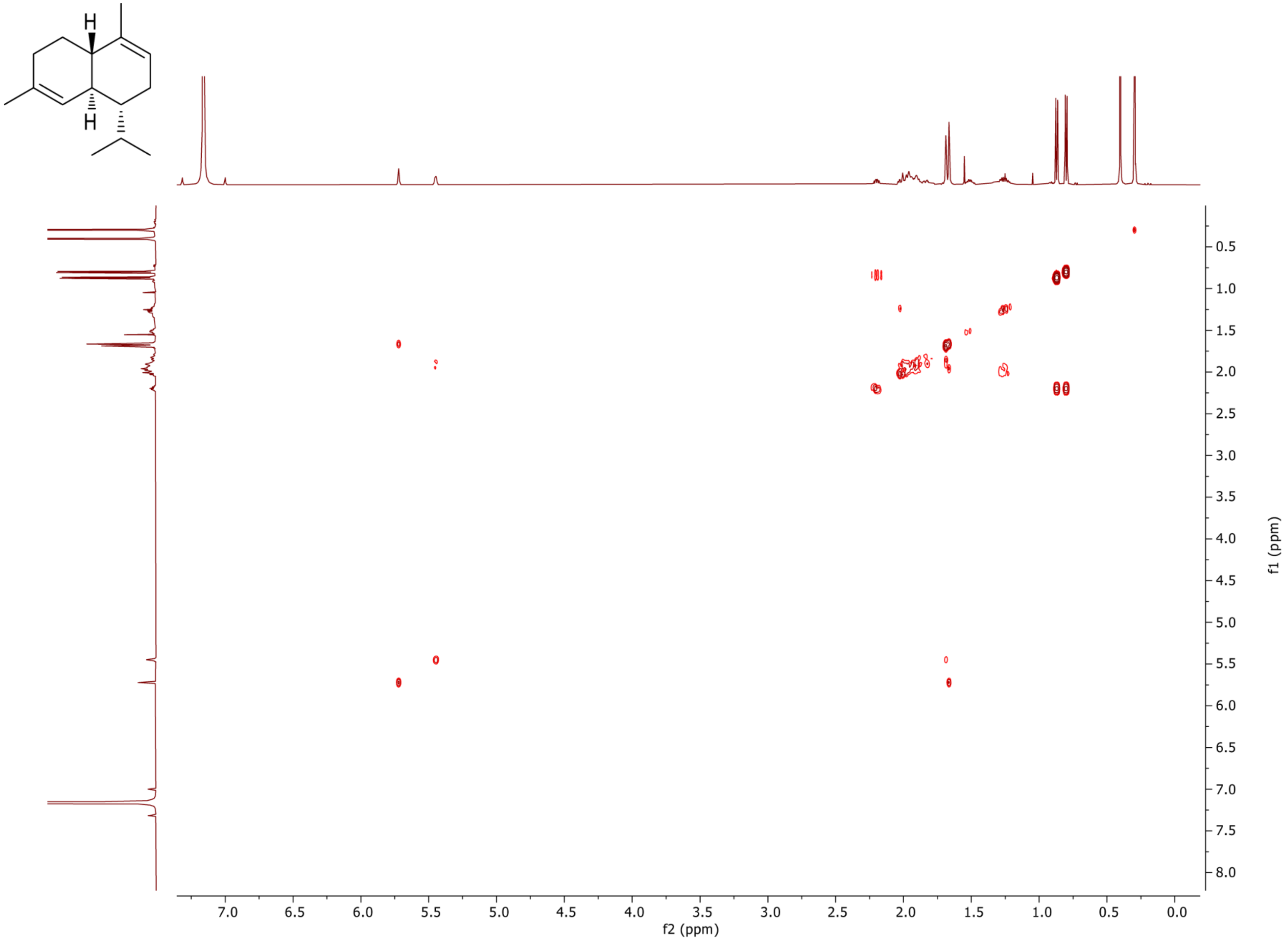

Figure S17. ^{1}H,^{1}H-COSY spectrum (C_6D_6) of **11**.

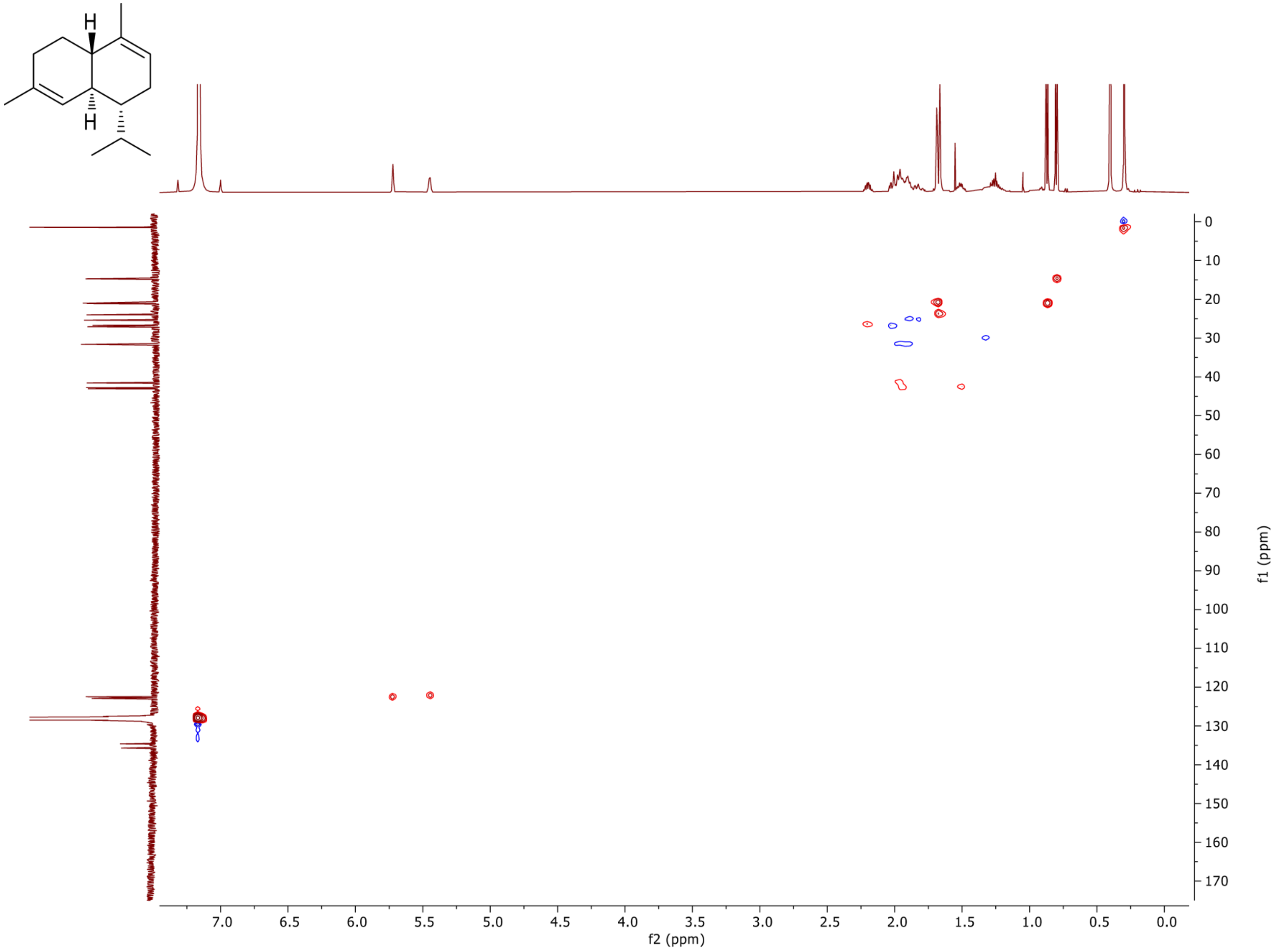

Figure S18. HSQC spectrum (C_6D_6) of **11**.

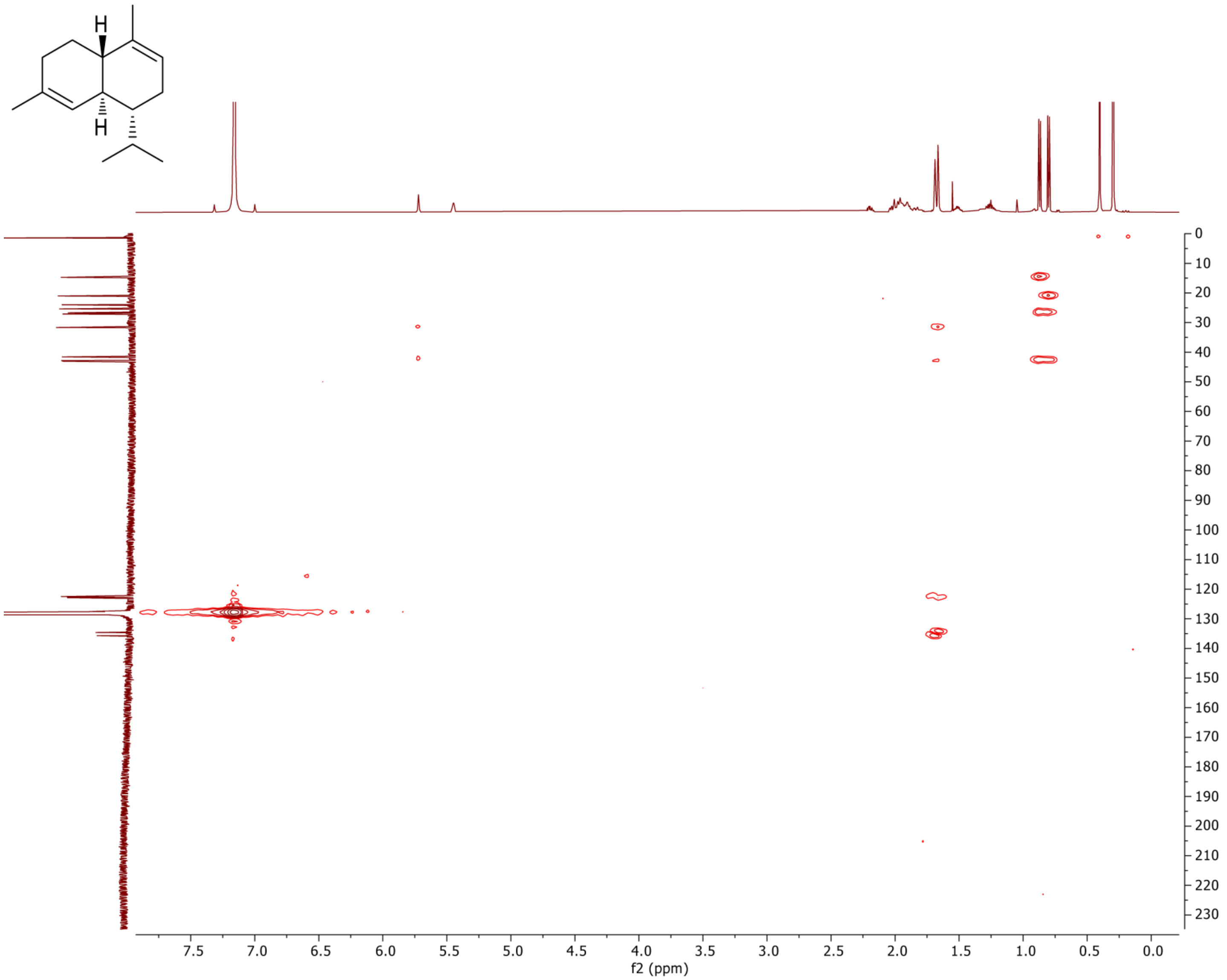

Figure S19. HMBC spectrum (C_6D_6) of **11**.

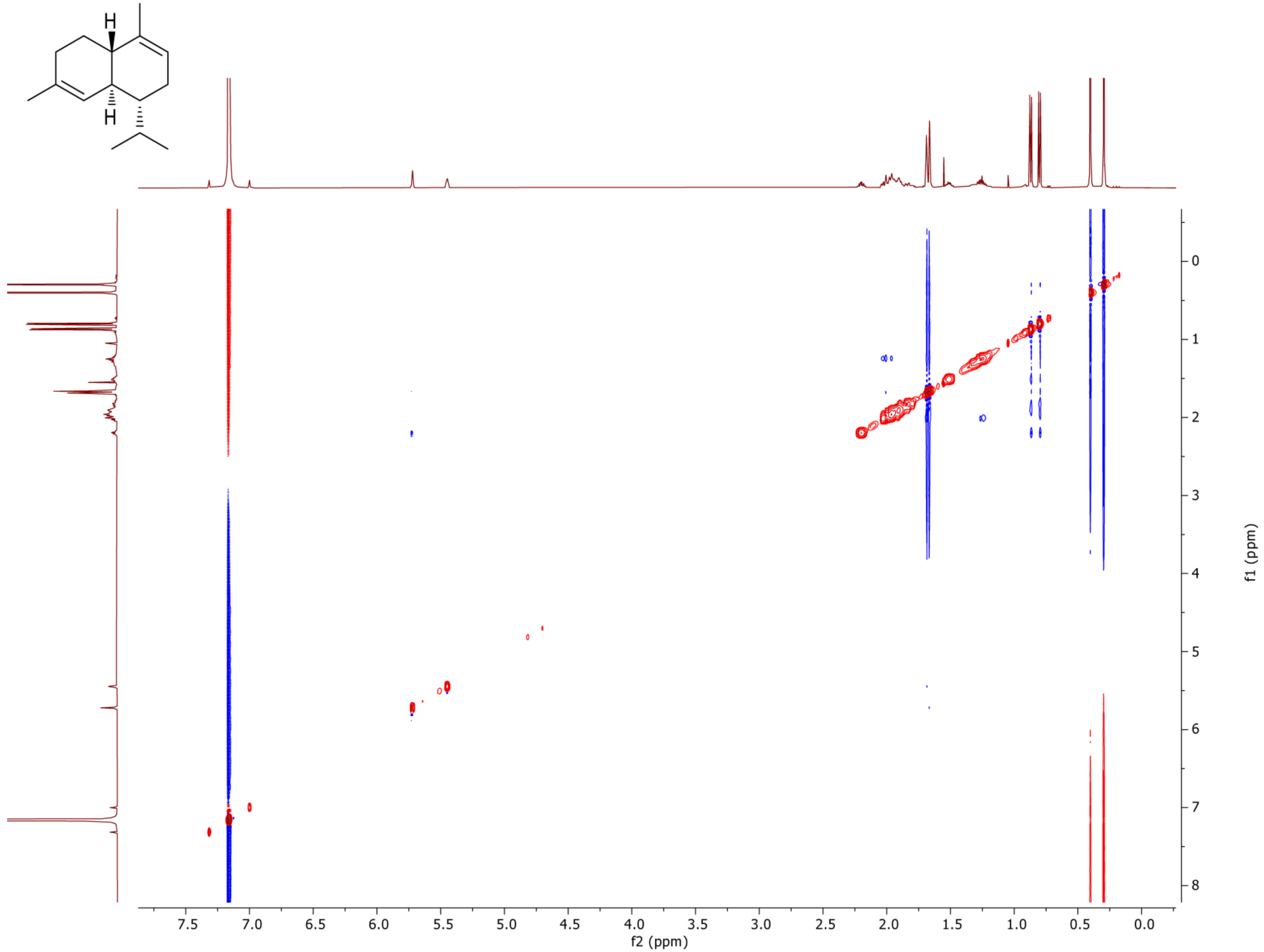

Figure S20. NOESY spectrum (C_6D_6) of **11**.

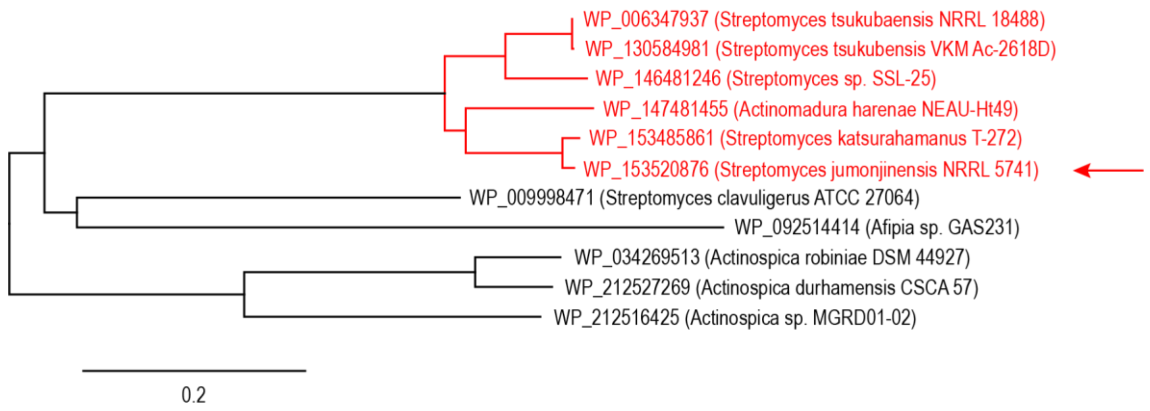

Figure S21. Detailed section of the phylogenetic tree shown in Figure 2 of main text. The red arrow indicates the characterised α-cadinene synthase (SjaCS). The closely related enzymes shown in red share a pairwise identity of 83% and may have the same function.

MTQLNSFSVPDFHLPFGNSKHPLAARANAEAATWAVRHELVTDAVDQFAGIGFGHLAGRVSG
DVPYETVVLLAEWMAWSFVLDDQHDHLIRTGEVAAWRPVAEAITEHLATGSAGGSGARRNPL
VKGFVDVCDRILDGMPEGPAARYRAHVPLMLDSLDQEAGNRGGEGRPTVRDYILMRRHSSQI
LPMMDMVEAGLGLDVPQRIHDLPEFKALVASALDVISWGNDVFSLPKEHSCGDNNNLVSLIS
SWEGCSLATAVRAVEGRIQDRIEEYLAGERLLTETLDARGETDPQVRAAVSRIVRSYEDWII
GADLWQRYECTRYSDERFAAGLESAYTRPGLVSVA

Figure S22. Amino acid sequence of SlADS (WP_078950427). Highly conserved residues are highlighted in yellow.

MTQLNSFSVPDFHLPFGNSKHPLAARANADATSWAVRHELVTDAVEQFAGIGFGHLAGRVSG
EVPYETVALLAEWMAWSFVLDDQHDHLIRTGELEAWRPVVGAITEHLETGGTPEAPGTPDTQ
GARRARQTQGARAAQGAGARRNPLVSGFVDVCDRILAGMSEGTAARYRAHVPLMLHSLDQEA
GNRGTVGRPSVDEYILMRRHSSQMLPMMDMVEAGLGIDLPQRIHDLPEFKALIASAVDVISW
GNDVFSLPKEYSCGDNNNLVSLIASWEGCSLADGVRAVENRIQARIEDFLTGERLLFETLDA
RGETDGAIRADVARCVRSYEDWIIGADLWQRYECTRYSDERFAAGLESAYTRPDLVWVA

Figure S23. Amino acid sequence of SsADS (WP_150516140). Highly conserved residues are highlighted in yellow.

Amorpha-4,11-diene (12). This compound was isolated by silica gel column chromatography with pentane. Yield: 1 mg, 4.9 μmol, 2.6%, from 80 mg (185 μmol) FPP triammonium salt. TLC (pentane): R_f = 0.69. GC (HP-5MS): *I* = 1480. Optical rotary power: $[\alpha]_D^{25}$ = –9.4 (*c* 0.64, CH_2Cl_2). The EI mass spectrum is shown in Figure 4B of main text and NMR data are given in Table S4.

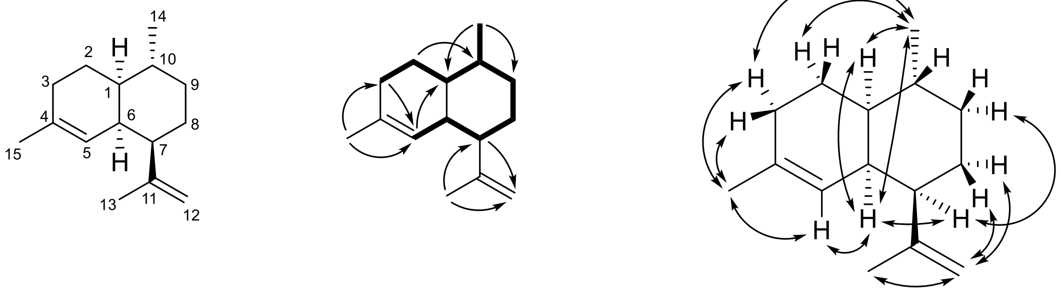

Figure S24. Structure elucidation of amorpha-4,11-diene (**12**). Bold: $^{1}H,^{1}H$-COSY, single headed arrows: key HMBC, and double headed arrows: NOESY correlations.

Table S4. NMR data of **12** in C_6D_6 recorded at 298 K.

		**12**	
C[a]	type	^{13}C[b]	^{1}H[b]
1	CH	42.11	1.18 (m)
2	CH_2	26.17	1.89 (m, H_α)
			1.51 (m, H_β)
3	CH_2	26.75	1.82 (m)
			1.69 (m)
4	C	134.61	–
5	CH	121.40	5.33 (br s)
6	CH	38.03	2.57 (m)
7	CH	47.97	1.92 (m)
8	CH_2	26.50	1.55 (m)
			1.36 (m)
9	CH_2	35.77	1.63 (m, H_β)
			0.93 (m, H_α)
10	CH	28.20	1.41 (m)
11	C	147.93	–
12	CH_2	110.39	4.99 (br s, H_E)
			4.81 (br s, H_Z)
13	CH_3	22.72	1.72 (s)
14	CH_3	20.10	0.87 (d, J = 6.5)
15	CH_3	23.86	1.61 (s)

[a] Carbon numbering as shown in Figure S24. [b] Chemical shifts δ in ppm; multiplicity: s = singlet, d = doublet, m = multiplet, br = broad; coupling constants *J* are given in Hertz.

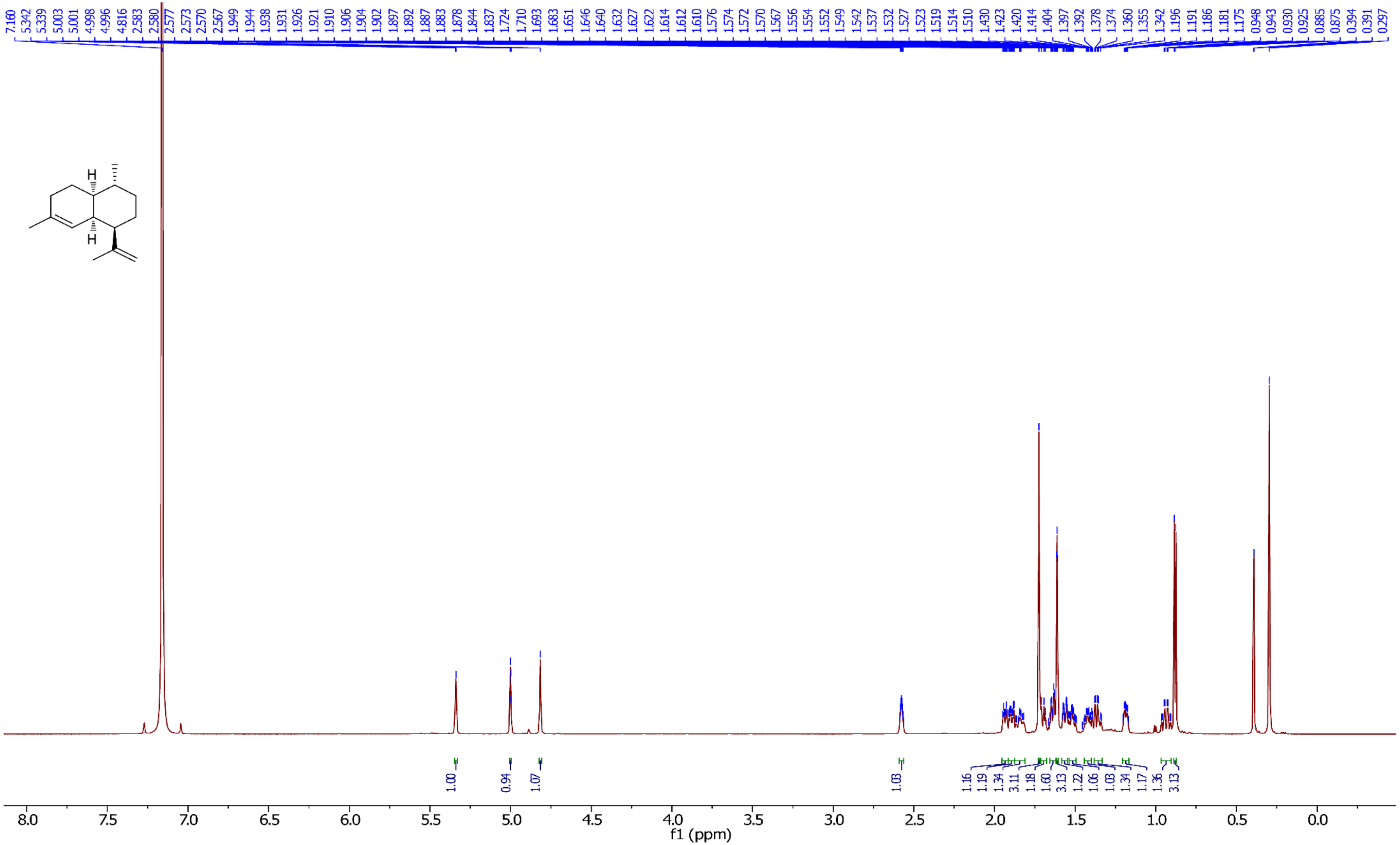

Figure S25. ^{1}H NMR spectrum of **12** (700 MHz, C_6D_6).

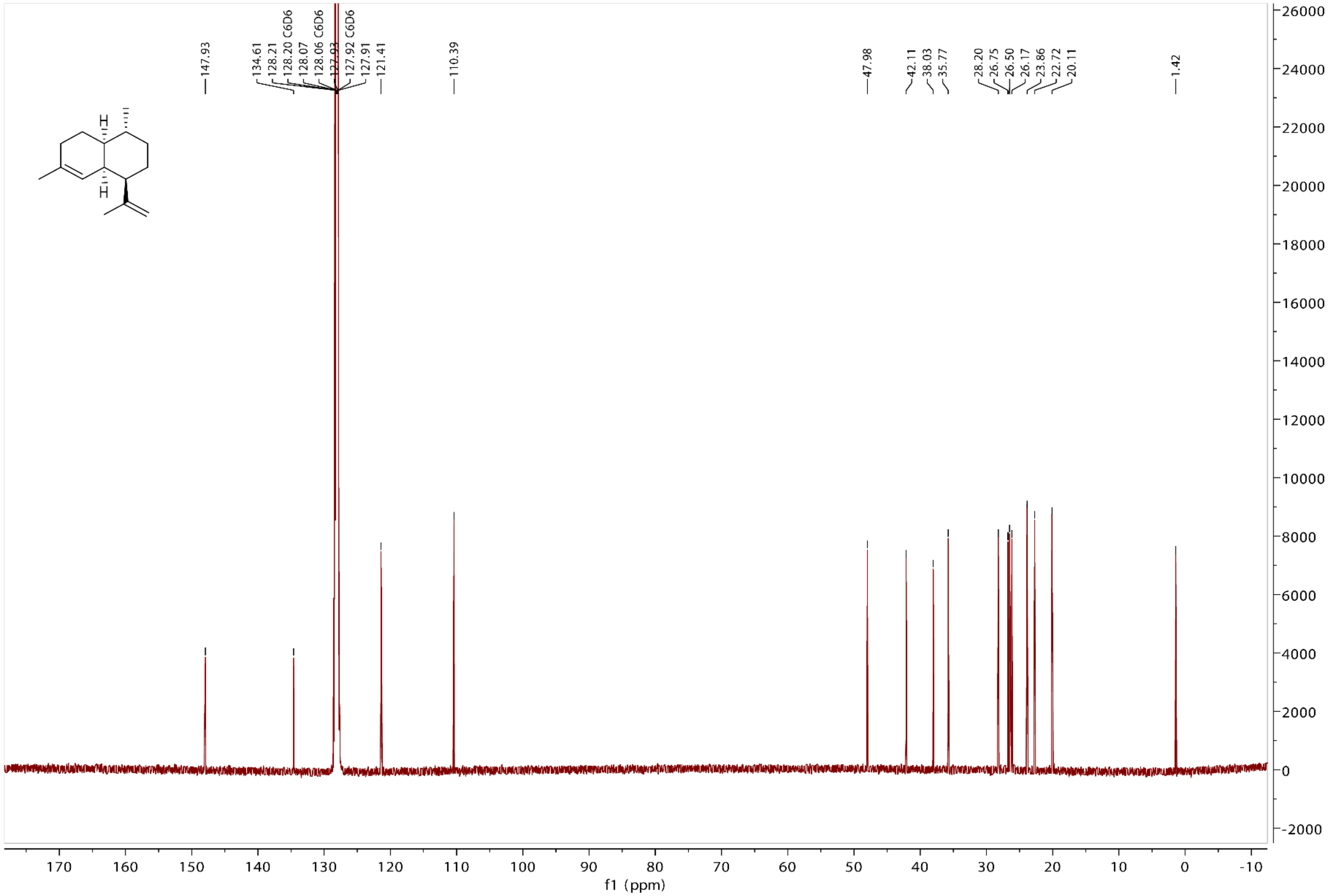

Figure S26. ^{13}C NMR spectrum of **12** (176 MHz, C_6D_6).

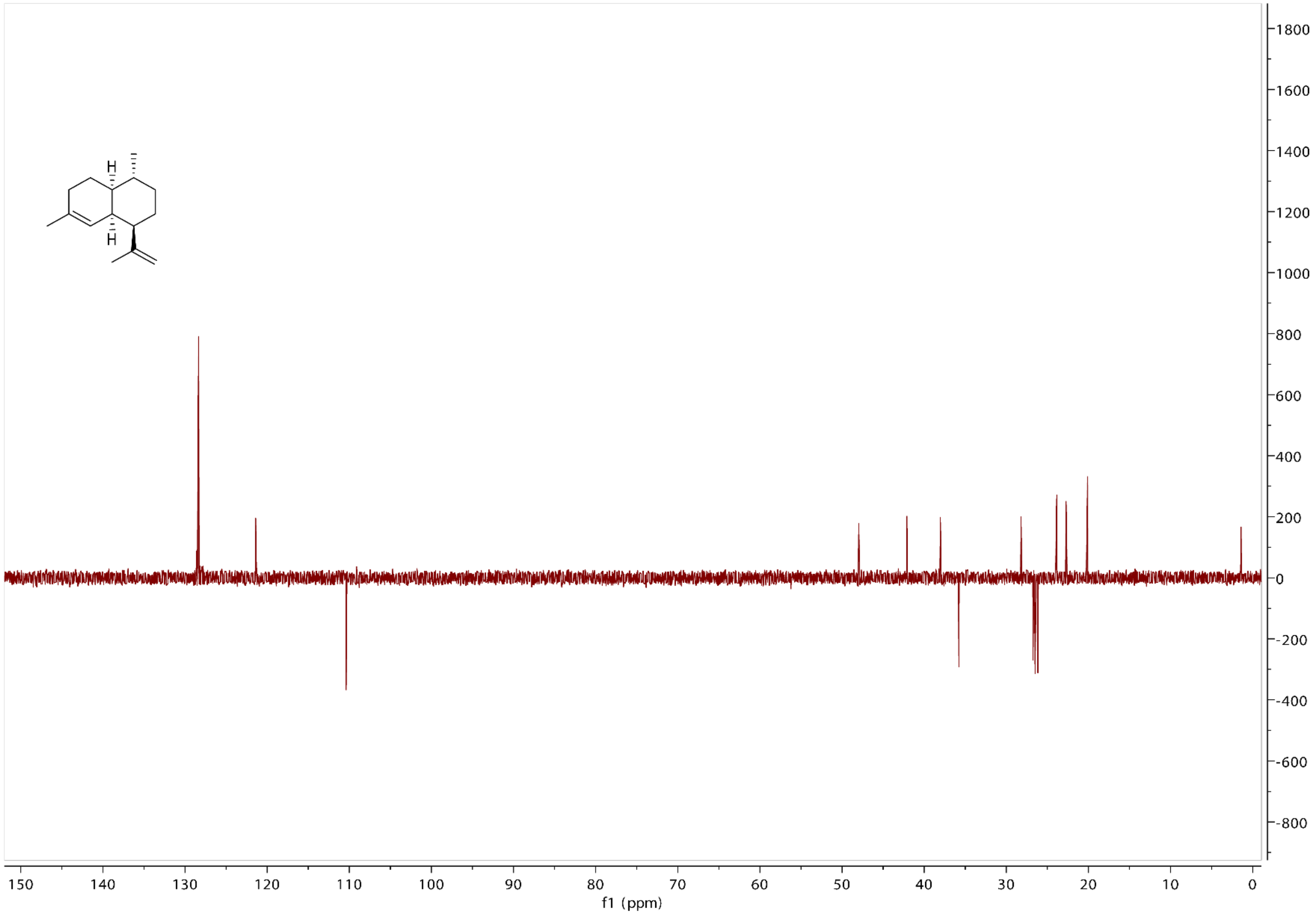

Figure S27. ^{13}C-DEPT135 spectrum of **12** (176 MHz, C_6D_6).

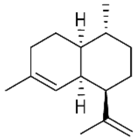

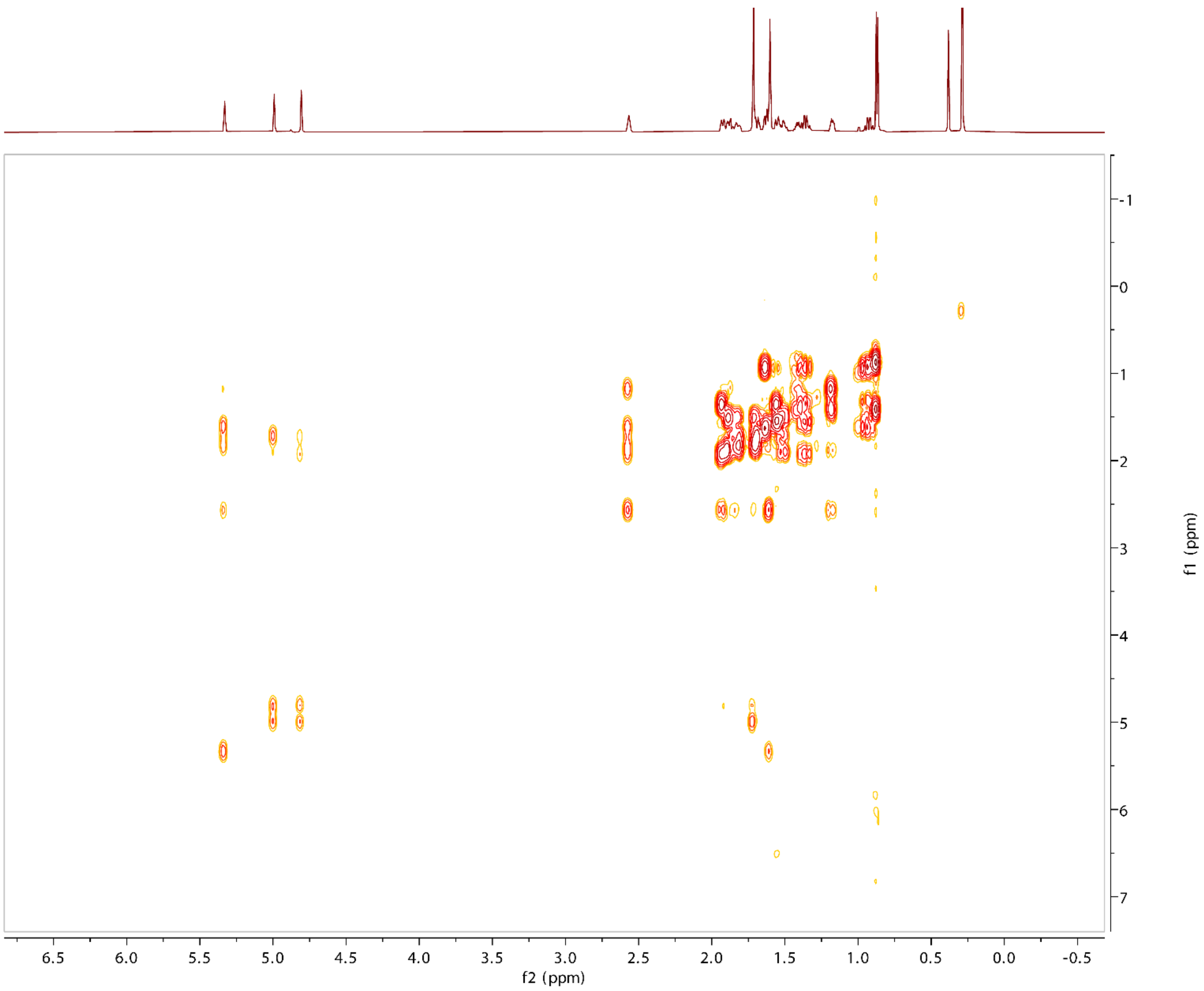

Figure S28. $^{1}H,^{1}H$-COSY spectrum (C_6D_6) of **12**.

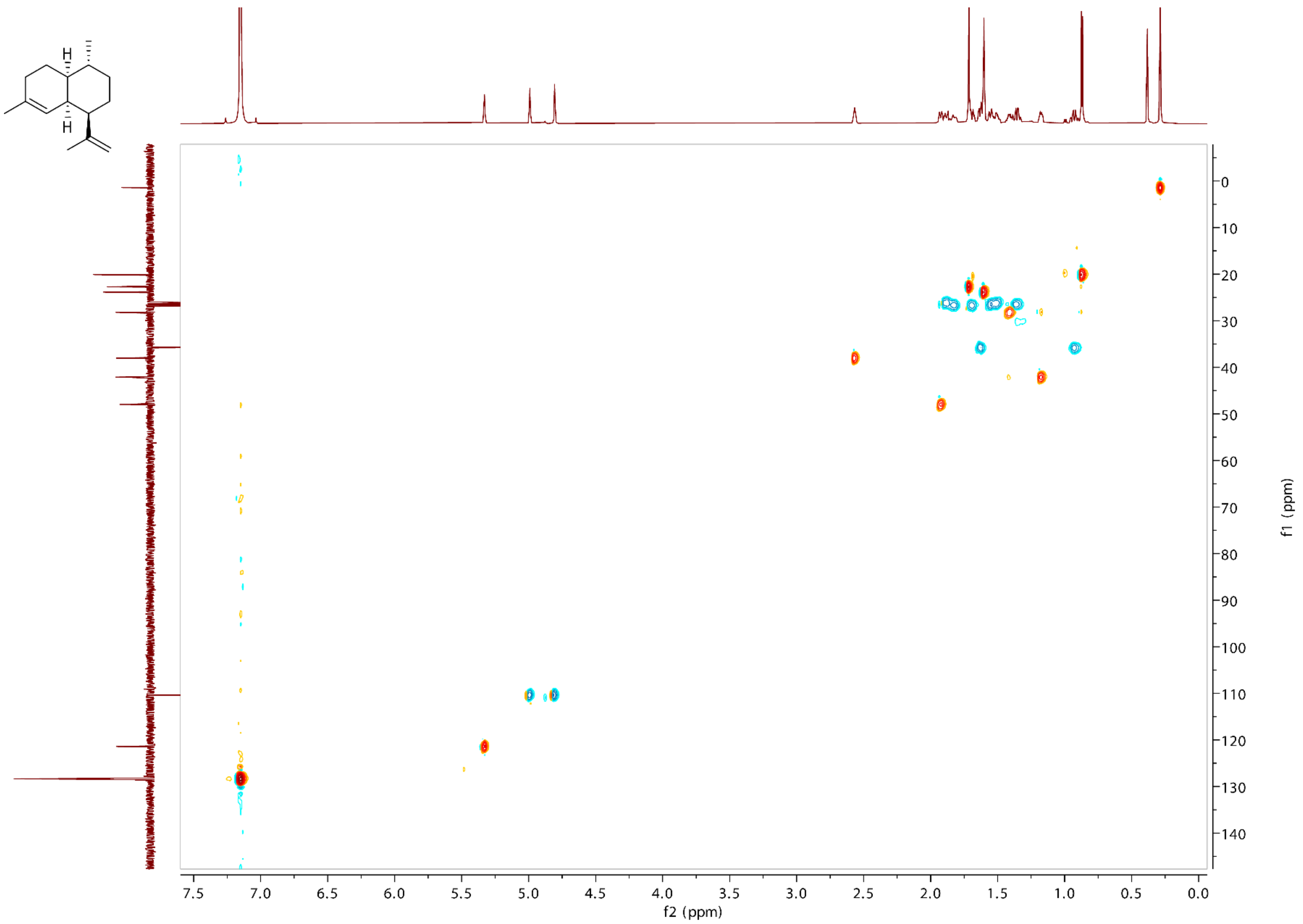

Figure S29. HSQC spectrum (C_6D_6) of **12**.

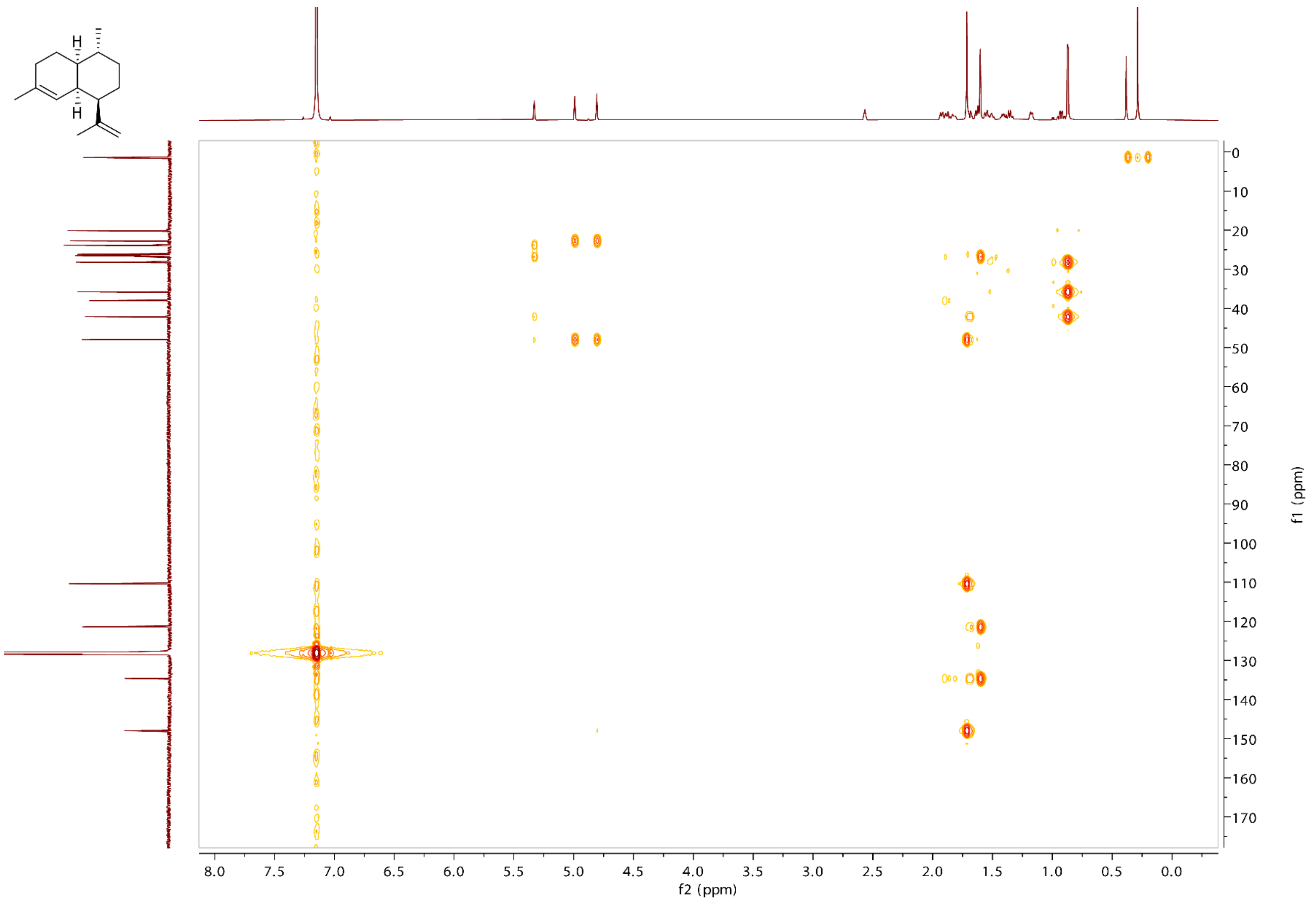

Figure S30. HMBC spectrum (C_6D_6) of **12**.

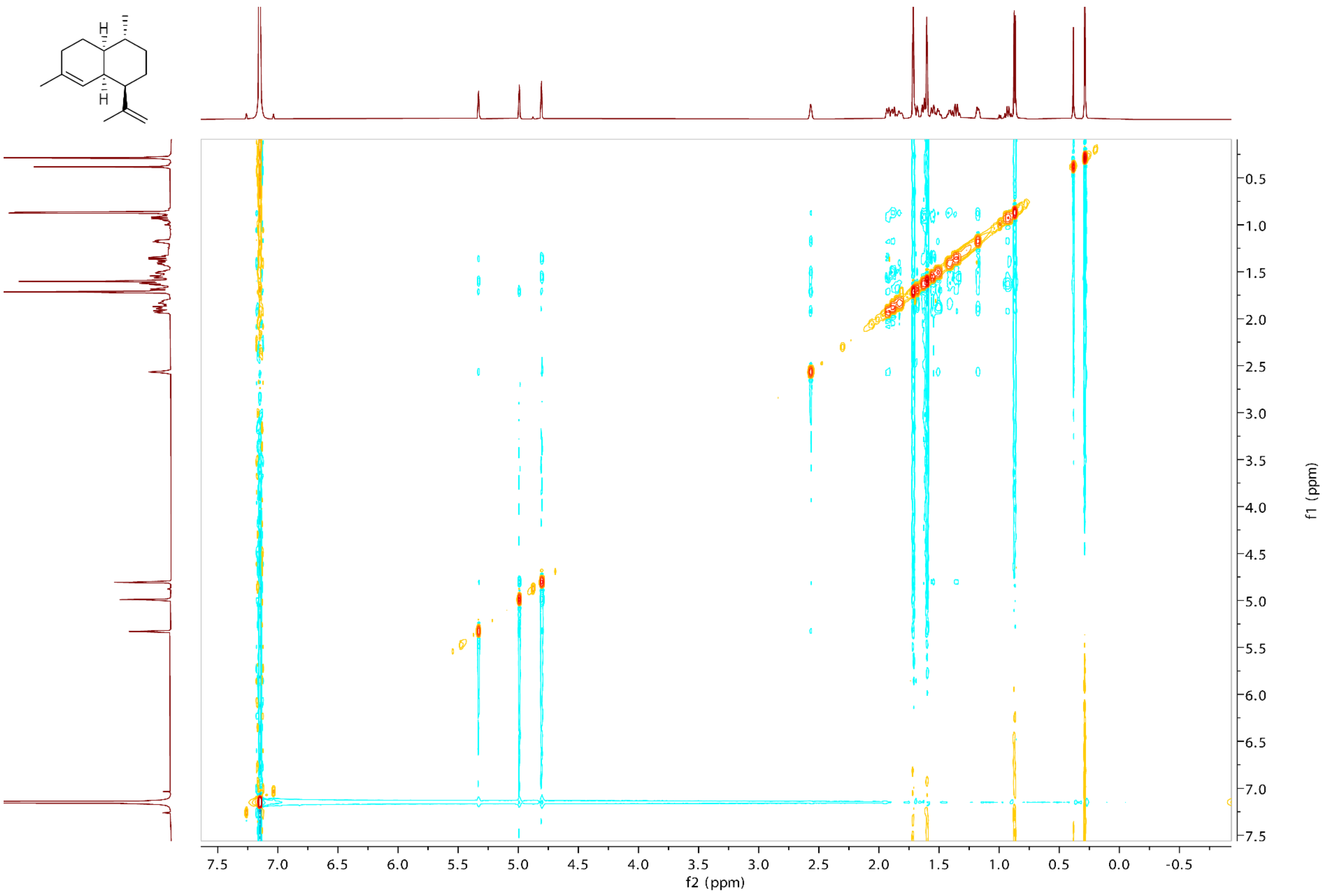

Figure S31. NOESY spectrum (C_6D_6) of **12**.

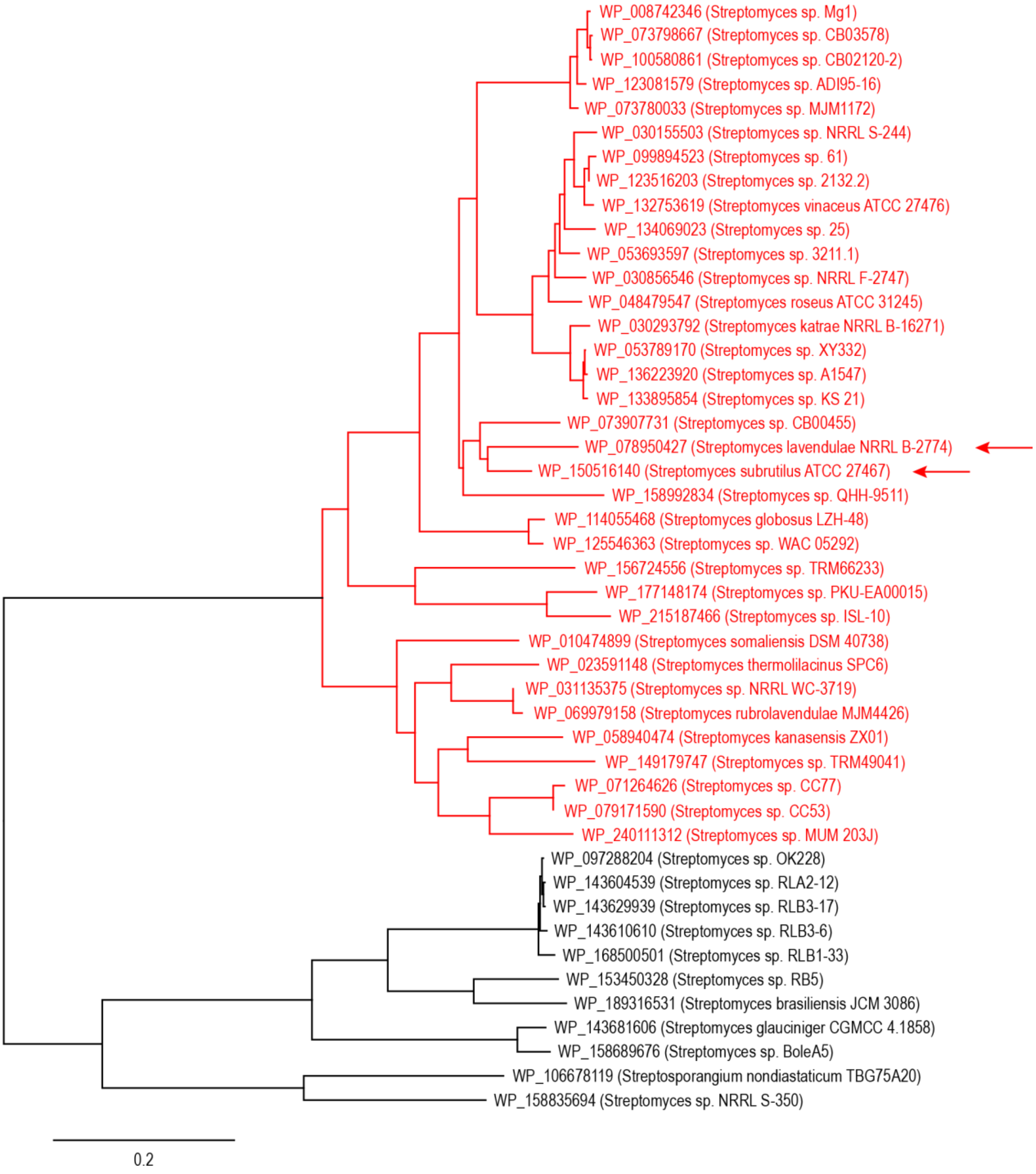

Figure S32. Detailed section of the phylogenetic tree shown in Figure 2 of main text. The red arrow indicates the characterised amorphadiene synthases (SlADS and SsADS). The closely related enzymes shown in red share a pairwise identity of 70% and may have the same function.

MTAVFERRHLPRIEMPFPRNRHRQQDLAQWHTRCWTVAHTLMKPDRATAYFDDLRYTDLIGG
YYVGAPLPVLNTINDFSLWFFVWDDRHNLDVVNRRQEGWSRLRDGLHAALDTPHRHINDPDP
LISAFCDCVVRFFEPFSDDWNARFITHFHSTIDAYDQEYRNRTTDSVPTVGDYLALRRHTFG
MWVWIDLLELAAGYELPSCVYSSSPYREAGLASQEFSAWYNDLHSMPKELAAGDFHNLGIVL
AHHEGLTVHEAAGEVARRIEQRIVNYRDHEGDVGQLLDDIGADPGLRAGVDRCLFNMRNWIS
SVYYFHDESSRYQLESWEDPARPLYVEEGREQ

Figure S33. Amino acid sequence of NbEIZS (WP_086008896). Highly conserved residues are highlighted in yellow. Sequence deviations within the conserved motifs are highlighted in green.

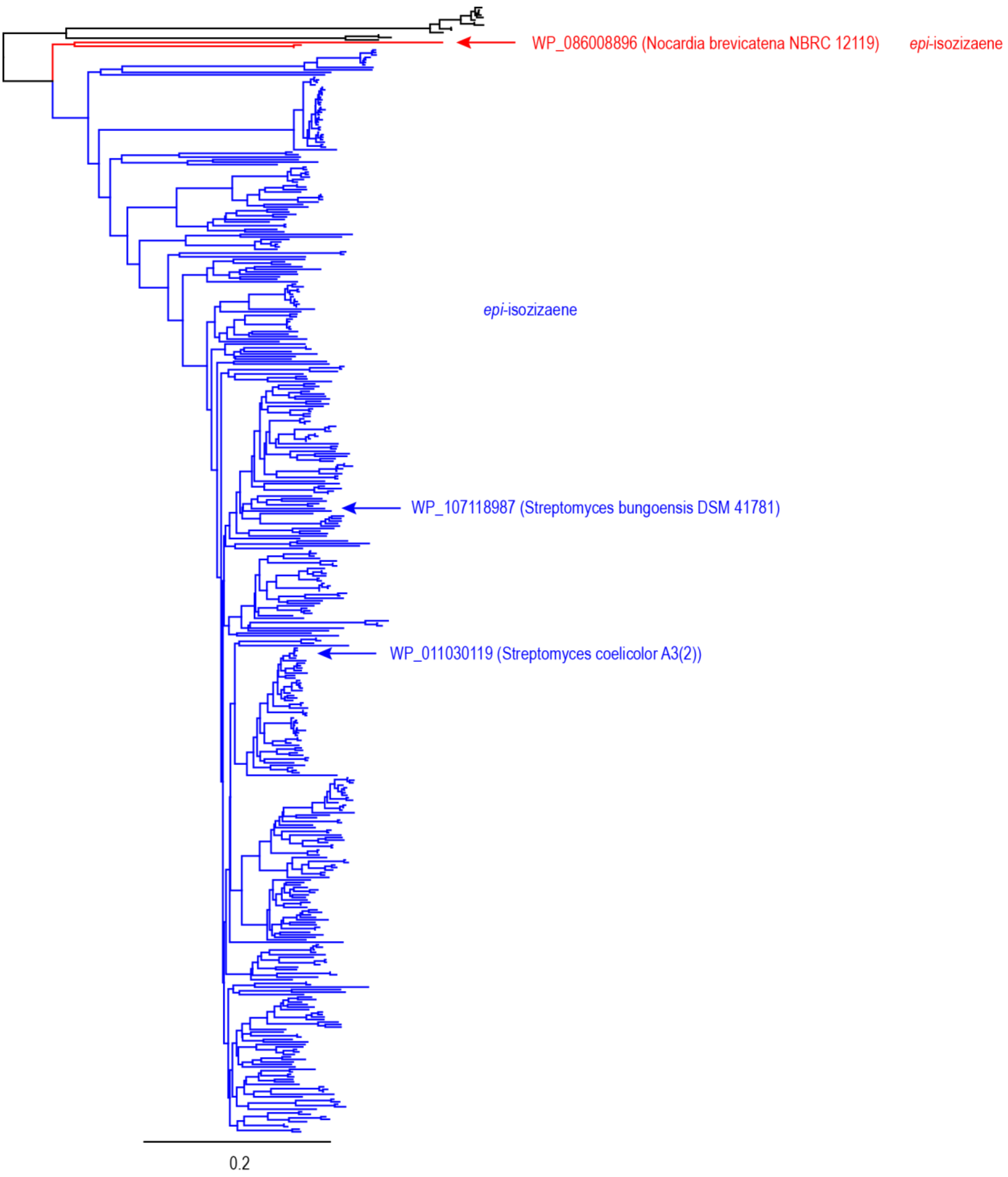

Figure S34. Detailed section of the phylogenetic tree shown in Figure 2 of main text. The blue arrows indicate the previously characterised *epi*-isozizaene synthases from *S. coelicolor* and *S. bungoensis*, and the red arrow highlights the *epi*-isozizaene synthase with a monoterpene synthase side activity from *N. brevicatena* (NbEIZS) characterised in this study.

Table S5. Identification of monoterpenes obtained with NbEIZS.

compound	I^a	I^a (lit.)	match[b]	reference
myrcene (**14**)	990	988	945	[6]
sylvestrene (**15**)	1029	1025	913	[6]
γ-terpinene (**16**)	1059	1054	905	[6]
cis-sabinene hydrate (**17**)	1067	1065	886	[6]
terpinolene (**18**)	1088	1088	928	[7]
linalool (**19**)	1099	1095	923	[6]
cis-*p*-ment-2-en-1-ol (**20**)	1122	1118	889	[6]
terpinen-4-ol (**21**)	1177	1174	896	[6]
α-terpineol (**22**)	1189	1186	893	[6]

[a]Retention index on a HP5-MS or (for the literature data) comparable GC column.
[b]Match factor of measured and data base mass spectrum (999 = identical mass spectra).

MPQDVRFDLPFTTPVSAHLEYAREQHLRWVRDMGLVRSQAGFEEYQSWDLPQAAIRTYPHAS
PDDMVVLMNWFSLAFLFDDQFDAASPDRADRVTEVARELIVTPLRPAGTRPRVICPITLAWA
EVWEQLSDGMSLTWCTRFAASWGRFLAAHAEEVDLAARGTLLGVGPYTAFRRRTVGIHHSID
AGERSRRFEVPAQAQAHPVMEGLRDAAADTIGFMNDIHSFEREKRRGDGHNLIAVLHRERGY
SWEKAAAEAFRMTVESLDTYVRLEARVPAMCGELGLDADGRDRVWMGVEAIRHWISGNYEWA
LTTGRYAAAKKGPAAAAELAGRGCLDDLLTV

Figure S35. Amino acid sequence of SfES (WP_028812116). Highly conserved residues are highlighted in yellow.

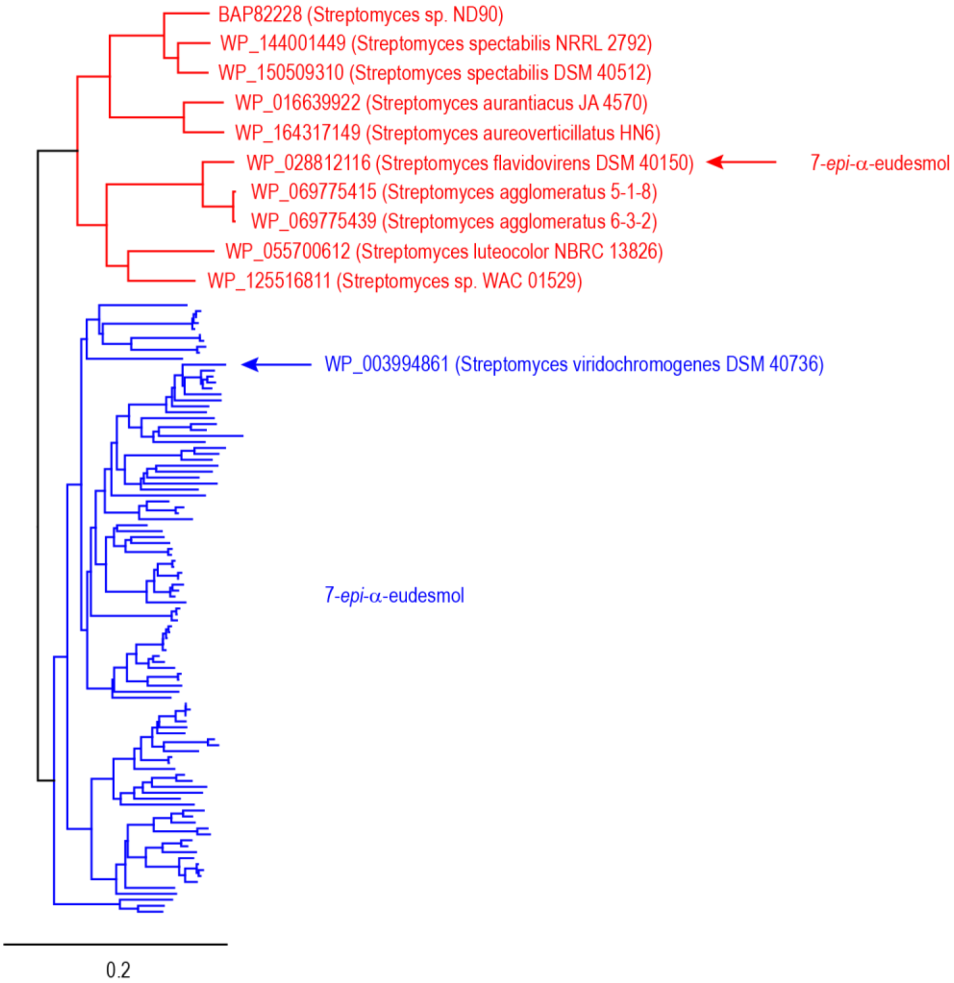

Figure S36. Detailed section of the phylogenetic tree shown in Figure 2 of main text. The blue arrows indicate the previously characterised 7-*epi*-α-eudesmol synthase from *S. viridochromogenes*, and the red arrow highlights the newly characterised 7-*epi*-α-eudesmol synthase from *S. flavidovirens* (SfES).

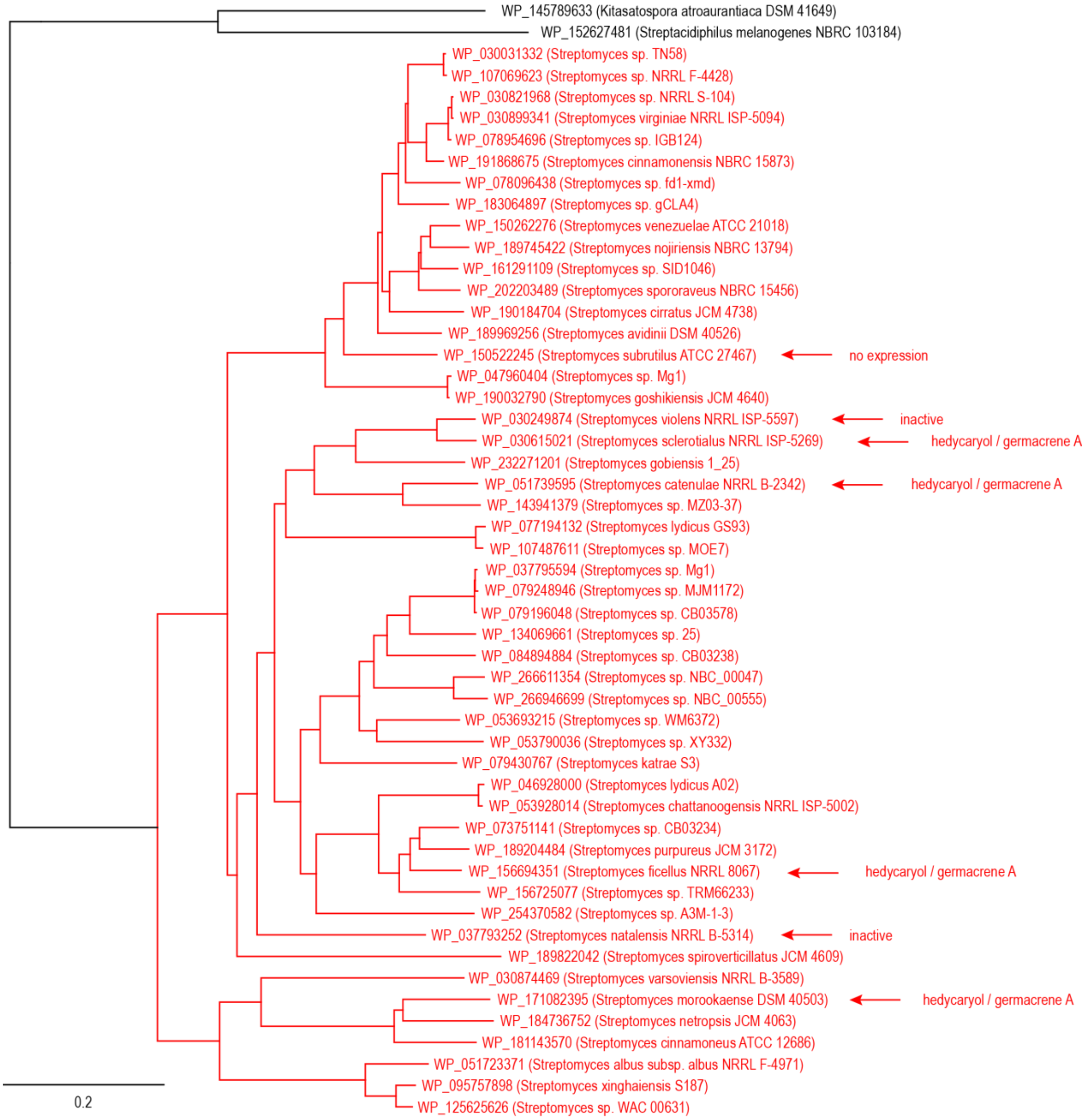

Figure S37. Detailed section of the phylogenetic tree shown in Figure 2 of main text. The red arrows highlight the newly characterised hedycaryol / germacrene A synthases from *S. sclerotialus* (SsHS), *S. catenulae* (ScHS), *S. ficellus* (SfHS) and *S. morookaense* (SmGAS).

MATITDRGRPSAIGWRLPPFYCPVERRTDLHAQADLLEKRAVEWIDAYGLYPDHTERAWGLA
THSAEFTCRIIPRGAEDAMLLFIQWNYWANAVDDWHDSGSDATGTAKIVDHSARLIRALEYP
GSAMLPSNPLTRALEDLVTRTRALLTPYQLMRFMDGTRDWLFGAGWQTSNAERGIMPSLNDF
AAMTVSTNGTRFTLAWCDAANAIDVPPDVLYSAPVQALTDAAGFVVSSDNDLFSYAKEDHLD
QPEQNLINVIAHEKGITPLEAVPYAVGIRDRAMTLFLRLREQLGRDADPMLARYLESLGDYV
SGCIIWQNDAPRYASPRNRNPLPVPGASYHVTYRDTPSDLATAPPAIPSITWWWDQLRD

Figure S38. Amino acid sequence of SsHS (WP_030615021). Highly conserved residues are highlighted in yellow. Sequence deviations within the conserved motifs are highlighted in green.

MTVTPDVRTTPGGDRSRPSPIGWQLPPFYCPITLTGDIHPRHAELERRALEWIDSYGLYPDA
TERAWGLATHSAEFSCRIIPHGEPEPLLIFMMWNYWAHAVDDWLDSGSNATATGKVVDTSIR
LLRALEYPGSAMMPPSPYTDALHDLVRRTRAALSPWQLRRFIDGIRDWLFASSWQTANAERG
VMPSLNDFAAMAVSINGTRFSLTWGEVANGADLPPDVLCSPAVQALTDAAGFLVGTDNDLFS
YAKEDHLEIPEQNLINVIAHERGCTPAEALPEAVGLRDRTMTLYLRLRDQLAADGDPMMRRY
LDTVGDYIAGCIVWQNNAPRFASPRNRNPLPVPGSSFGITYRDTPSDPSTDAPALPAIAWWW
DQLTSPDAP

Figure S39. Amino acid sequence of ScHS (WP_051739595). Highly conserved residues are highlighted in yellow. Sequence deviations within the conserved motifs are highlighted in green.

MPPSYAEDTPPSRIGWRLPPFYCPFDRDLLHPKAAELEERAVAWIDAYGLYPDATERAWGLA
THSADFTSRIIPHGDVEPILLFIEWNYWANAVDDWQDSGSAATRTADITDHSARLVRTIEAP
GSRLLPRGPLTEALDDLVSRTRAMLTPFQLRRFTEGTRDWLFGAGWQTANTERDVMPSLNEF
AAMRASVNGTRFTLTWCDAANDIRLPADVLYSAPVQALTDAAGFIVSCDNDLFSYNKEDHQE
PREQNLLNVVARDRGCTPREALVPAVALRDRAMTLFVRLSEQLARDAGEPLRRYLDSLAHYV
AGCITWQNRAPRYASPRNRNALPVEGASYDITWRDTPSDPSPEPPPVPAIAWWWRQLDG

Figure S40. Amino acid sequence of SfHS (WP_156694351). Highly conserved residues are highlighted in yellow.

MTDTSTRPRPSAIGWRLPPFYCPIEPAVHPRAGQLEQRAIAWLDSRGIFGNARDRAWSIATH

STDFSCRIIPYGDDEPLLLFIEWNHWAFALDDICHDTGSADIRTATIVDLNARIARCLEVPG

SGMLGSSPFDAALEDLAARTRAMTTPVQLRRVTEGLRDWLFGAAWQVSNVERGVMPSLSDYV

AMRPSINGTRFSLSWSEIAGGIRIPDHELYSAPVQALTEAAGFIVSCDNDLFSYAKEDSQET

TDQNIVNVLAHERRCPAEQALEEALVIRDRAMTLFLDLRAQIARNAGLHLRRYLEALGHYIS

GCIRWMDAAPRYASPRNRYDLPVPGATYGITWRDSPRDTGTEPLPIASAAWWWEQLDAPVTA

CAATA

Figure S41. Amino acid sequence of SmGAS (WP171082395). Highly conserved residues are highlighted in yellow.

MTDDQLLVELPPRYCPVQVDTHPDAELLEGQGADWLTGYGLAGPRLRANDCAGFYGRIMPKA
VTERLQMAVDWCFLMFAFDDINCDEVVPDAAGKQFVDIATRVVRVLEVPDVNLGPTADLFLA
PVRDLALRAHRFGTPTQVRRLVDGHRAWYLGVLWELRCKLADVTPSLNDYAHMRQHTAGGLA
TTSWIEIVDGAEIPAAELDSPAVRALSELAFTIAAWDDDLFSYGKETWFARRETPSNCKLNL
IDIVALERGYSVEAALVESVELVNRLTHRFIQLRDAVLPSASEELRNYLECLSRLLRGNMEW
GLQAQRYRNPDGRSPDAVRTVGSWTEEPPAHADVVPAIPSIAWWWDPELGL

Figure S42. Amino acid sequence of KkAS (WP_184867163). Highly conserved residues are highlighted in yellow. Sequence deviations within the conserved motifs are highlighted in green.

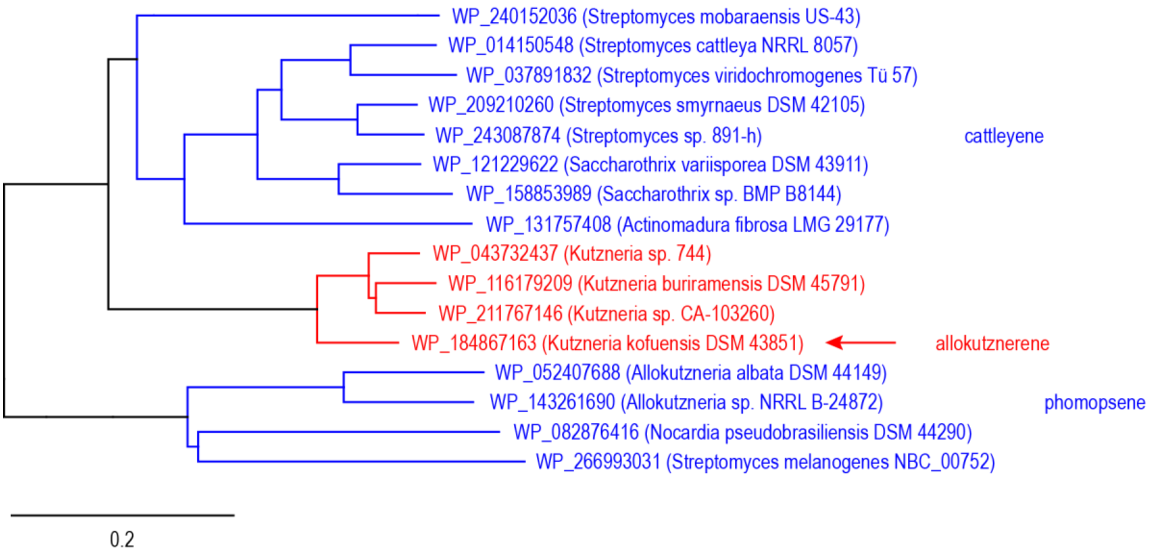

Figure S43. Detailed section of the phylogenetic tree shown in Figure 2 of main text. The red arrow highlights the newly characterised allokutznerene synthase from *K. konfuensis* (KkAS).

MNLADLPEGFWTFYCPLDEETGADAERLSANSAAWAQKFDLGLGDANLASLYGAGGASLITH
AFPHATTDPDLAQALADYSAWAFMTDDFIVPDPNARADILHTVYRWAHTMQVPRSWESQGTH
LDDALRNVLERLRACMSDVQYERFTTAQAGWLHAMLWERALRERGTALTVNDYLAVRIGAVG
VHATLGYLDAVEGTEITAQEWSSPPVKAAVEASLFAAALDNDRYSFCKESDLAQVNYNLFGA
LQHEHPDWTLAQAMIEGIAIRDTMLALYLRLREQILPTASPDLRKYLTGVERVVSGDITFGT
TCMRYFAPEAAPHIQRTFTPPAHLSDEPLPYPTIAWWWEHITP

Figure S44. Amino acid sequence of sesterviolene synthase from *Streptomyces* sp. Tü 2975 (WP_159685978). Highly conserved residues are highlighted in yellow. Sequence deviations within the conserved motifs are highlighted in green.

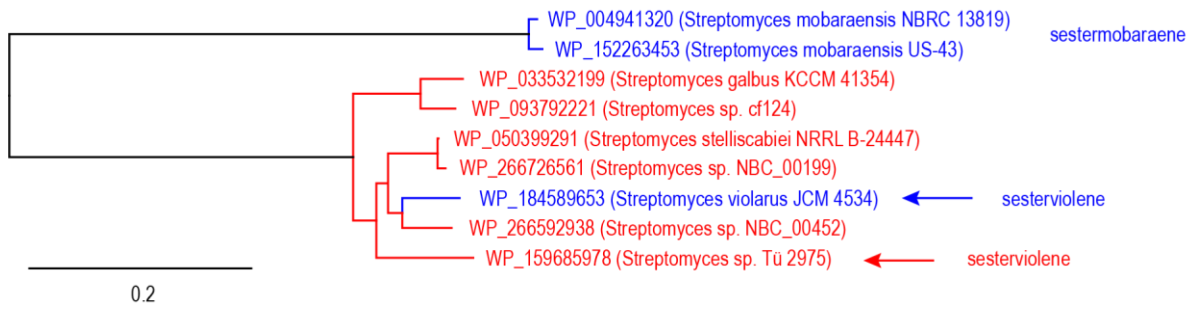

Figure S45. Detailed section of the phylogenetic tree shown in Figure 2 of main text. The red arrow highlights the newly characterised sesterviolene synthase from *Streptomyces* sp. Tü 2975 (StSS) and the blue arrow indicates the known enzyme SvSS from *Streptomyces violarus* with same function.

References

[1] Neumann, B.; Pospiech, A.; Schairer, H. U. *Trends Genet.,* **1992,** *8,* 332–333.

[2] Dickschat, J. S.; Pahirulzaman, K. A. K.; Rabe, P.; Klapschinski T. A. *ChemBioChem,* **2014,** *15,* 810–814.

[3] Giets, R. D.; Schiestl, R. H. *Nat. Protoc.,* **2007,** *2,* 31–34.

[4] Bradford, M. M. *Anal. Biochem.,* **1976,** *72,* 248–254.

[5] Fulmer, G. R.; Miller, A. J. M.; Sherden, N. H.; Gottlieb, H. E.; Nudelman A.; Stoltz, B. M.; Bercaw, J. E.; Goldberg, K. I. *Organometallics,* **2010,** *29,* 2176–2179.

[6] Adams, R. P., *Identification of Essential Oil Compounds by Gas Chromatography / Mass Spectrometry,* Allured Business Media, Carol Stream, USA, **2009**.

[7] Adams, R. P. *Biochem. Syst. Ecol.,* **1999,** *27,* 709–725.
